# Effects of Home-Based Interval Walking Training on Thigh Muscle Strength and Aerobic Capacity in Female Total Hip Arthroplasty Patients: A Randomized, Controlled Pilot Study

**DOI:** 10.1371/journal.pone.0108690

**Published:** 2014-09-30

**Authors:** Yutaka Morishima, Takashi Mizushima, Katsuya Yamauchi, Mayuko Morikawa, Shizue Masuki, Hiroshi Nose

**Affiliations:** 1 Department of Sports Medical Sciences, Shinshu University Graduate School of Medicine, Matsumoto, Japan; 2 Department of Rehabilitation, Hamamatsu University School of Medicine, Hamamatsu, Japan; 3 Jukunen Taiikudaigaku Research Center, Matsumoto, Japan; Tokyo Institute of Technology, Japan

## Abstract

Due to the reduced physical activity of patients who have undergone total hip arthroplasty (THA), there are no home-based exercise training regimens for preventing muscle atrophy and aerobic capacity impairment in these patients. We examined whether interval walking training (IWT) could prevented these issues. Twenty-eight female patients (∼60 years of age) who had undergone THA more than 2 months prior were randomly divided into IWT (n = 14) and control (CNT, n = 14) groups. The IWT subjects trained at a target of 60 min of fast walking at >70% peak aerobic capacity for walking (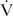
O_2peak_) per wk for 12 wk, while those in the CNT maintained their previous sedentary life during the same period. We measured the energy expenditure of the daily physical activity, except during sleeping and bathing, every minute and every day during the intervention. We also measured the isometric knee extension (F_EXT_) and flexion (F_FLX_) forces, 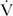
O_2peak_, and anaerobic threshold during the graded cycling exercise (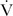
O_2AT_) before and after the intervention. All subjects, except for one in IWT, completed the protocol. F_FLX_ increased by 23% on the operated side (*P* = 0.003) and 14% on the non-operated side of IWT (*P* = 0.006), while it only increased on the operated side of CNT (*P* = 0.03). The 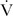
O_2peak_ and 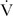
O_2AT_ in IWT increased by 8% (*P* = 0.08) and 13% (*P* = 0.002), respectively, and these changes were significantly higher in the IWT than in CNT group (both, *P*<0.05). In conclusion, IWT might be an effective home-based training regimen for preventing the muscle atrophy from reduced daily physical activity in THA patients.

**Trial Registration:**

UMIN-CTR UMIN000013172

## Introduction

Total hip arthroplasty (THA) is a broadly prescribed surgical treatment for patients with advanced arthritic disorders of the joint. [1] However, THA patients are thought to suffer from muscular atrophy and weakness on the operated limb side more than 2 years after surgery; [2], [3] these changes might be accompanied by joint instability, prosthesis loosening, risk of falls, and other complications, [4]–[7] and they might advance to impaired mobility of the joint, [1], [8]–[11] resulting in reduced daily physical activity and aerobic capacity in a vicious cycle.

To prevent these problems, exercise training for rehabilitation is performed immediately after surgery under the supervision of medical staff while the patient stays in the hospital with financial support from national insurance; however, a maximum period of 2 wk is recommended for hospital management reasons, and this period is too short for recovering muscle strength. Patients are allowed to visit a hospital for rehabilitation up to several months following discharge, and resistance training using machines has also been reported to be effective; [12] however, limited financial support from insurance, impaired mobility, and the inconvenience of the transportation options for travelling to the hospital might discourage patients from continuing exercise training [13]–[16].

Therefore, the development of a home-based exercise regimen is needed so that THA patients can perform habitual exercise to increase their muscle strength on the side of the treated limb. Recently, we developed an exercise training system for middle-aged and older people featuring 1) interval walking training (IWT), 2) the use of a portable calorimeter, and 3) the e-Health Promotion System. Using the system, we found that middle-aged and older people who performed IWT for 5 months had ∼10% increased thigh muscle strength and peak aerobic capacity for walking (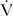
O_2peak_) [17], [18] with a much higher adherence rate to IWT (94%) than for a standard walking training regimen (∼60%); however, there have been no studies on THA patients [19].

Therefore, the purpose of this pilot study was to examine whether the training system is helpful for increasing thigh muscle strength and aerobic capacity as well as improving daily physical activity in THA patients.

## Methods

The protocol for this trial and supporting CONSORT checklist are available as supporting information; see Checklist S1 and Protocol S1.

### Ethics Statement

This study was approved by the Ethics Committee of Hamamatsu University School of Medicine and was in accordance with the Declaration of Helsinki. All participants provided written informed consent for participation.

### Trial registration

The clinical trial was registered after participant recruitment for this study. However, we recruited the first participant for this study after approval by the ethics committee of Hamamatsu University School of Medicine. Additionally, all ongoing and related trials for the exercise training of THA patients are registered.

### Participants

The criteria for subjects with total hip arthroplasty were that they were able to walk independently without any assistive devices. The exclusion criteria were suffering from any acetabular and/or femoral prosthesis failure or other comorbidities or the presence of any cardiopulmonary, neurologic, or cognitive diseases.

**Figure 1:**
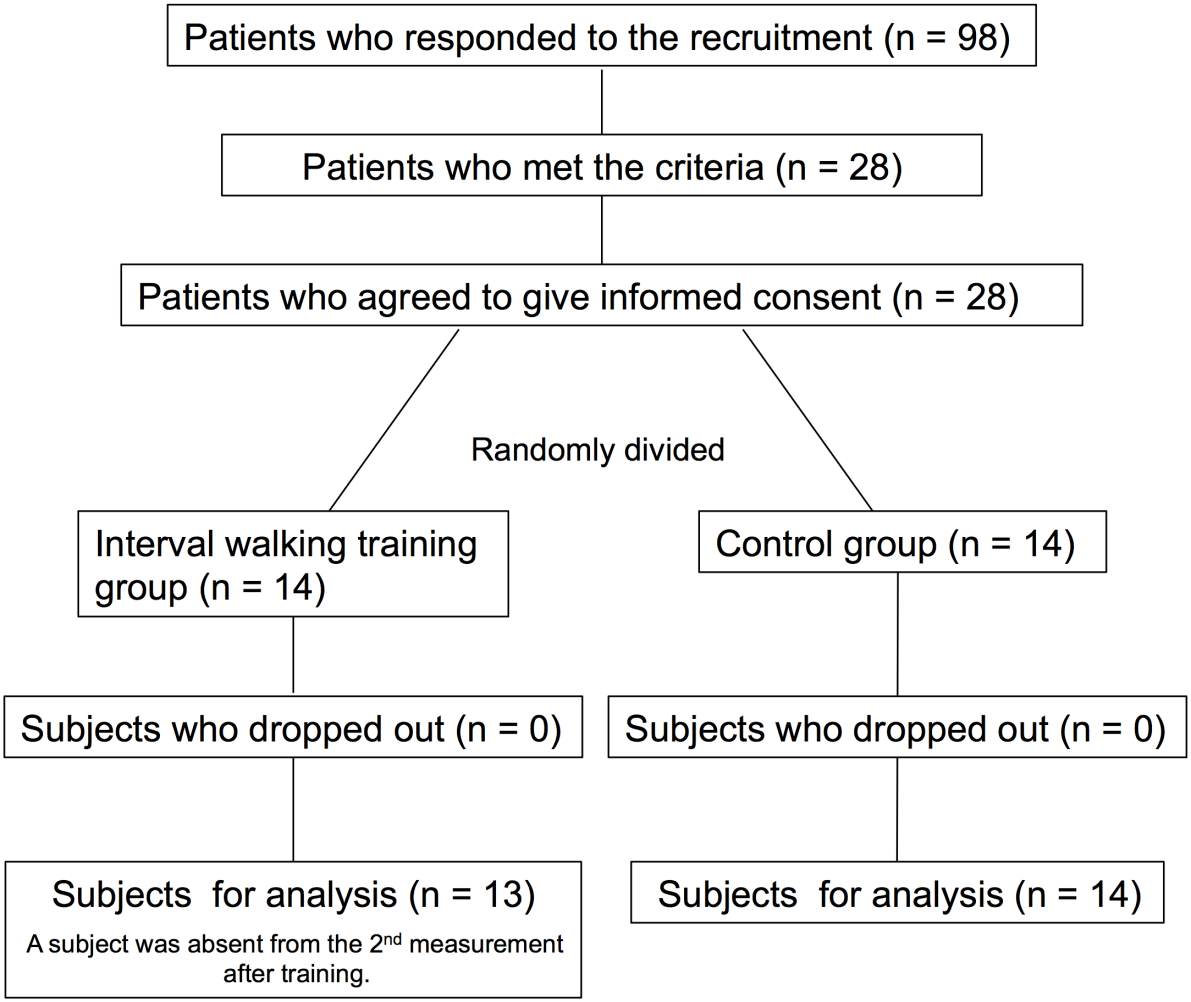
CONSORT flow diagram. Flow profile of the subjects.

**Figure 1** shows the flow profile of subjects in the present study. Recruitment and screening began on August 1. To recruit subjects for this study, we mailed leaflets to female patients who had undergone THA at Hamamatsu University Hospital and who visited the hospital for rehabilitation before June 2009. As a result, 28 of 98 respondents met the criteria, and all provided written informed consent before participating in the study. They were randomly divided [20] into interval walking training (IWT, n = 14) and control (CNT, n = 14) groups such that there were no significant differences in the medical history, current physical characteristics (**Table 1**), and other measurements before training.

**Table 1:** Physical characteristics of the subjects.

	CNT (n = 14)	IWT (n = 13)	*P* (Group)
Age (years)	59.9±5.4 (52–68)	60.3±7.4 (52–74)	NS
Height (cm)	151.9±5.8 (144–166)	153.3±3.4 (146.8–159.4)	NS
Body mass (kg)	57.0±9.6 (48.0–69.8)	54.0±5.3 (43.3–80.6)	NS
BMI (kg/m^2^)	24.7±4.1 (20.1–35.4)	23.0±2.6 (19.4–28.5)	NS
Reason for operation	OA: 13; ANFH: 1	OA: 13	NA
Operated side(left/right/bilateral)	7/4/3	5/5/3	NA
Postoperative period (months)	34±46 (2–170)	58±58 (9–181)	NS

Values are given as the means ± SD (range). CNT, control group; IWT, interval walking group; OA, osteoarthritis; ANFH, avascular necrosis of femoral neck head; BMI, body mass index; and NA, not applicable.

### Randomization

Allocation was performed using permuted-block randomization (block of 4). YM generated the random allocation sequence. Each eligible and consenting subject was assigned to one of the groups. Only known one researcher (YM) knew of the exact allocation.

### Intervention Protocols

The training experiments were conducted from September 6, 2009 to December 20, 2009. The subjects were instructed to visit the gym at 8∶30 am on the assigned day in September after overnight fasting. After interviewing the subjects about their current hip joint pain, their subjective feeling of satisfaction with walking using the visual analogue scale (VAS) and their quality of life with the Medical Outcomes Study 36-item short-form health survey (SF-36), [21], [22] we measured their height and body weight. After allowing them to eat a light breakfast and rest for an hour, we measured the thigh muscle strength and 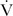
O_2peak_. Within a week, the subjects were invited to our laboratory in the hospital an hour or more after breakfast or lunch and were asked to participate in a measurement of the anaerobic threshold by graded cycling exercise (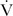
O_AT_). [23] When they left the laboratory after the measurement, we provided them with a portable calorimeter with a tri-axial accelerometer (JD-Mate; Kissei Comtec, Matsumoto, Japan) and instructed them to measure their energy expenditure due to physical activity every minute during the day, except during sleeping and bathing periods, for 7 consecutive days before the start of training.

Before IWT, the subjects visited a gym near the hospital and received instructions about the exercise program. They were told to repeat 5 or more sets of 2- to 3-minute low-intensity walking intervals at ∼40% of the pre-training 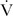
O_2peak_, followed by a 3-minute interval of high-intensity walking at >70% but <85% 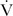
O_2peak_, ≥4 days/wk; the total fast walking time per week reached ≥60 min. The intensity and steps were monitored with JD-Mate, which was worn on the mid-clavicular line of the right or left waist. A beeping signal alerted subjects when a change of intensity was scheduled, and another sound told them when their walking intensity had reached the target level. Once subjects had learned the program, they were allowed to choose their training time. Additionally, in IWT, the energy expenditure (oxygen consumption rate) due to physical activity other than IWT and steps was measured with JD-Mate during the day, except for during bathing and sleeping periods, while the beeping signal and sounds of the device were switched off. The total energy expenditure due to physical activity is presented in **Table 2**, including that for IWT.

**Table 2:** Total energy expenditure for IWT and physical activity.

	CNT (n = 14)			IWT (n = 13)					
	Before	During	After	Before	During	After	*P*	*P*	*P*
	(1 wk)	(12 wk)	(1 wk)	(1 wk)	(12 wk)	(1 wk)	(Group)	(Time)	(Group×Time)
§Total energy expenditure	14614±1496	13616±1361	10258±1827	14032±1133	14744±1012	13824±1495	NS	NS	NS
(O_2_ ml/kg/wk)									
Total steps	50537±5411	51636±5411	40159±6749	50693±5845	53016±5845	44619±6105	NS	NS	NS
(steps/wk)									
Fast walking									
§Intensity	10.6±0.6	12.0±1.4	9.7±1.3	9.7±0.6	8.9±0.9	10.2±0.8	NS	NS	NS
(O_2_ ml/kg/min)									
Time	76±16	69±15	75±17	100±27	156±15* †	127±18†	<0.0001	<0.0001	<0.0001
(min/wk)									
§Energy expenditure	786±196	805±153	710±176	828±211	1363±157†	1221±169†	.013	NS	NS
(O_2_ ml/kg/wk)									

Values are given as the means ± SE. Before, for 1 wk before training; During, for 12 wk during training; and After, for 1 wk after training.

§ Resting oxygen consumption is not included.

*Significant difference from the value before training, *P*<0.05;

† Significant difference from the corresponding value in CNT, *P*<0.05. The other abbreviations are the same as in **Table 1**.

Every 2 weeks, the subjects visited the hospital, and data from the tracking devices were transferred over the internet to a central server in the administrative center for automatic analysis and reporting of the IWT effects from a database of more than 3,000 middle-aged and older people without THA; we call this system the e-Health Promotion System (Kissei Comtec). [24] Physical therapists used these reports to track the daily walking intensity and the other parameters given in **Table 2** to instruct subjects on how best to achieve the target levels. If the targets were not met, they encouraged subjects to increase their efforts to achieve them.

Subjects in CNT were instructed to maintain the same lifestyle as before training. Every 2 weeks during the training period for IWT, they visited the hospital, and the energy expenditure due to daily physical activity and steps during the day, except during sleeping and bathing periods, was transferred over the internet from the tracking devices to the central server in the administrative center, but, unlike in IWT, there was no automatic analysis and reporting.

After the 12-wk training period, we measured the same variables as before training for both groups. After the post-training measurements, we instructed the subjects in both groups to continue measuring their energy expenditure from daily physical activity and steps during the day, except during the sleeping and bathing periods, for 7 consecutive days after the end of training. The data were then transferred to the server after measurements.

### Number of subjects for the analyses

In IWT, one subject was excluded from the following analyses because she was absent on one of the measurement days assigned after training. Therefore, we analyzed 13/14 and 14/14 subjects in IWT and CNT, respectively.

### Measurements

#### Thigh muscle strength

Isometric bilateral knee extension (F_EXT_) and flexion (F_FLX_) forces were measured with an isometric dynamometer (GT-330; OG Giken, Tokyo, Japan). The higher value from 2 trials each for extension and flexion forces, which were displayed on the screen of the dynamometer, was adopted for analysis. Similarly, we measured the strength of the other knee joint.

Because 3/13 subjects in IWT underwent THA on both hip joints and the remaining 10 subjects underwent THA on one side, we analyzed the effect of IWT on the thigh muscle strength on 16 operated sides and 10 non-operated sides in this group. Similarly, in CNT, 3/14 subjects underwent THA on both hip joints, and the remaining 11 subjects underwent THA on one side. Therefore, we analyzed the thigh muscle strength before and after training for 17 operated sides and 11 non-operated sides in this group.

#### 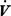 *O_2peak_*

Subjects with the accelerometer on their back walked for 3 minutes on a flat floor at 3 graded, subjective velocities (slow, moderate, and fast) with ∼25°C room temperature and ∼60% relative humidity. At the same time, the heart rate was recorded with an electrocardiogram, and 3-dimensional acceleration was measured at 10-millisecond intervals and recorded as averaged values of the 5-second memories. [25], The total impulse from the accelerometer was transferred to a computer and converted to the oxygen consumption rate (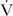
O_2_) using a previously reported equation. [25], [26] The 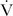
O_2peak_ and peak heart rate (HR_peak_) for walking values are those for the last 30 seconds at the maximal walking speed.

#### 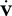
O_2AT_

We determined the 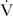
O_2AT_ at ∼25°C room temperature and ∼60% relative humidity with an expired gas analysis system (AE-310S; Minato, Tokyo, Japan). The 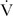
O_2_ and carbon dioxide production (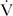
CO_2_) were measured for every breath. The exercise intensity was increased by 10 watts per minute and stopped when the anaerobic threshold was judged to have been reached according to the standard method [23], [27], [28] by viewing the 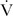
O_2_ vs. VCO_2_ relationship displayed on the screen of the system during exercise. We also determined the heart rate at 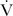
O_2AT_ (HR_AT_).

#### Total energy expenditure due to IWT and physical activity in daily life

As shown in Table 2, the total energy expenditure due to physical activity was determined by adding the 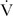
O_2_ (O_2_ ml/kg/min) before (1 wk), during (12 wk), and after (1 wk) the training periods after excluding the energy expenditure at rest, which was assumed to be constant during these periods, and this energy is presented as the O_2_ ml/kg/wk. Similarly, the walking steps were determined by summing the steps for the respective periods and are presented as steps/wk. A 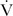
O_2_ above 70% W
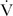
O_2peak_ for individuals was regarded as the intensity for fast walking (O_2_ ml/kg/min), and the total energy used for fast walking was determined by summing the 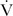
O_2_ for the respective periods, after excluding the rate during rest, and this energy is presented as O_2_ ml/kg/wk.

#### Questionnaires

Hip joint pain was evaluated with the 100-mm VAS scale, where 0 represents “no pain” and 100 represents the “worst pain”. Similarly, walking satisfaction was evaluated using 0 as “no satisfaction” and 100 as “full satisfaction”. The SF-36 was used to evaluate the quality of daily life [21], [22].

### Statistical Analysis

**Figure 2:**
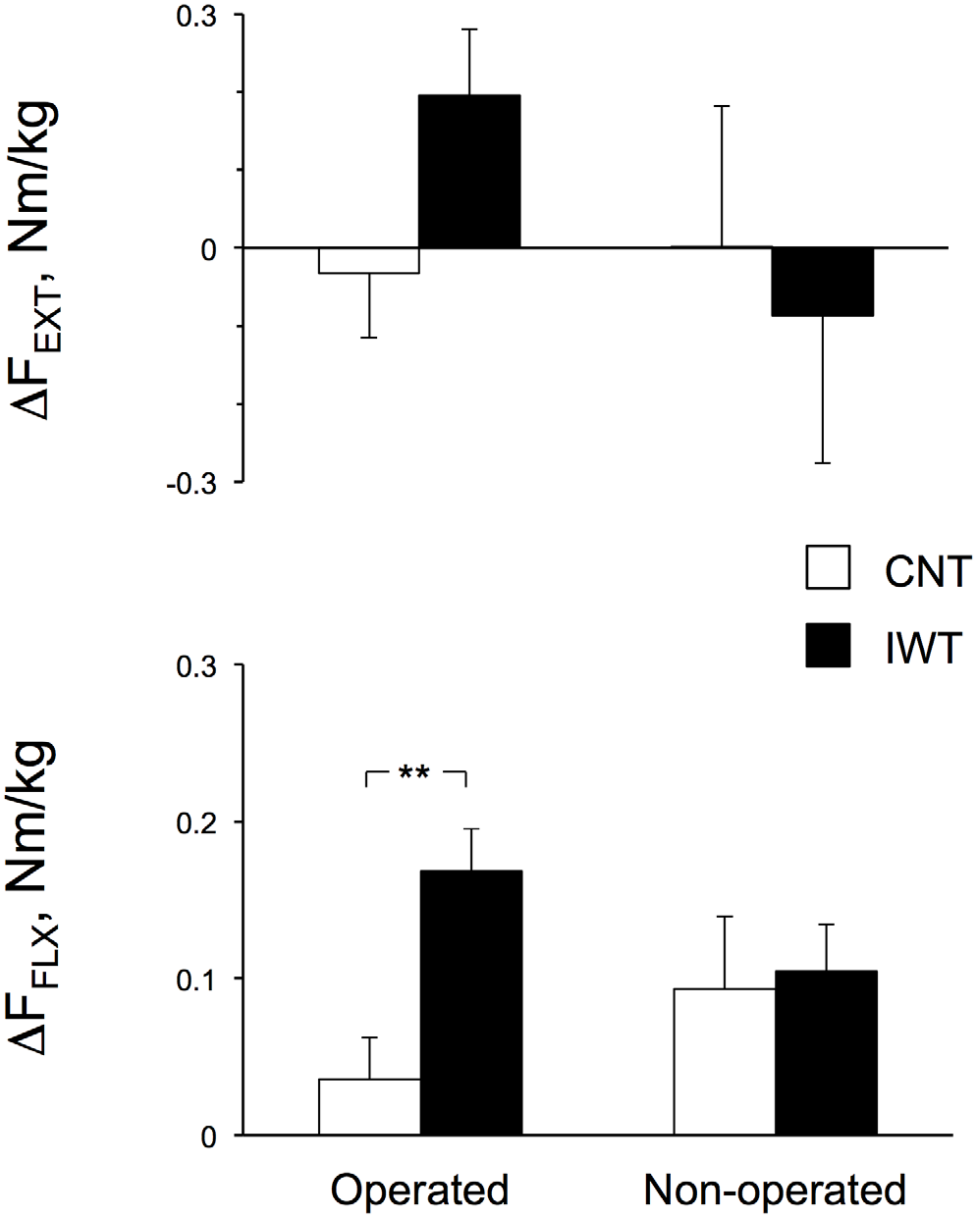
Changes in the thigh muscle strength after training on the operated and non-operated sides. The isometric knee extension (F_EXT_) and flexion force (F_FLX_) are shown as the means with SE bars for 17 and 16 measurements on the operated side and 11 and 10 measurements on the non-operated side in 14 and 13 subjects in the control (CNT) and interval walking training (IWT) groups, respectively. Significant difference between the CNT and IWT groups, **P<0.01.

The Mann-Whitney *U* test was used to test for differences in the physical characteristics between the IWT and CNT groups (**Table 1**). Two-way analysis of variance (ANOVA) [group (CNT vs IWT)×time (before vs. after training)] for repeated measures was also used to examine any significant differences in the variables before and after training between groups (**Tables 2** **–** **4** and the questionnaires). The model was also used to examine any significant changes in the variables after training with an interactive effect of (group×time) on the variables (**Tables 2** **–** **4** and the questionnaires). After confirmation of the significance by ANOVA, the Tukey-Kramer method was used as a post-hoc test to examine any significant differences in variables in specific comparisons between groups before and after training (**Tables 2** **–** **4** and the questionnaires). We determined significant differences in the changes of the variables after training between groups by ANCOVA after correcting for their pre-training values as covariates (**Figure 2** **&** **3**). We present the statistical power (1-β) in the text at α = 0.05 in both groups when the variables increased significantly after training or when the increases were significantly greater in IWT than in CNT with the minimum sample sizes to detect the greater increases (**Figures 2** **&** **3**). All data are reported as the mean±standard error (SE), unless otherwise indicated. The null hypothesis was rejected at *P*<0.05.

**Figure 3:**
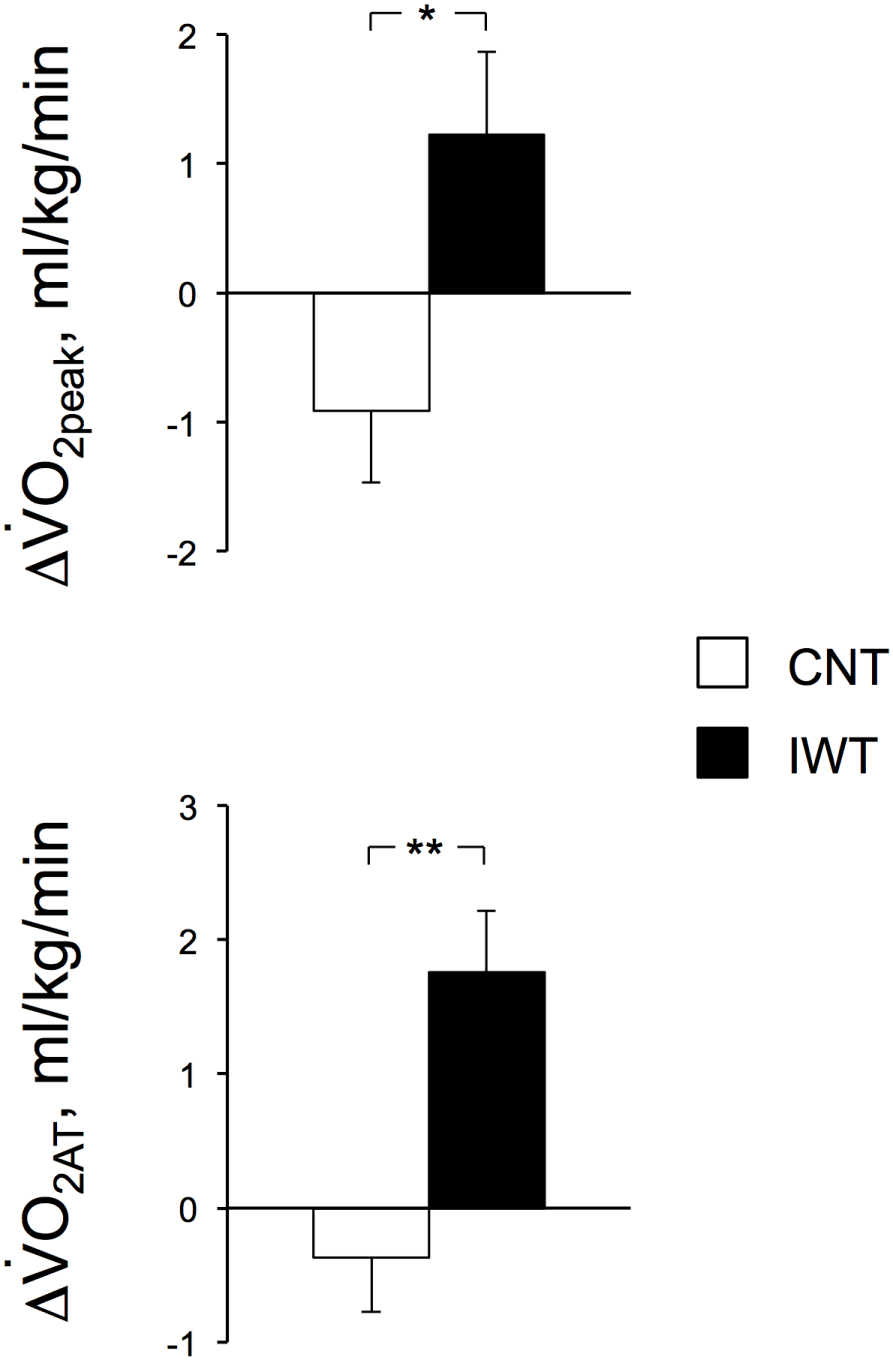
Changes in the aerobic capacities after training. The peak aerobic capacity for walking (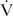
O_2peak_) and anaerobic threshold by graded cycling exercise (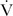
O_2AT_) are shown as the means with SE bars for 14 and 13 subjects in the CNT and IWT groups, respectively. Significant differences between the CNT and IWT groups, *P<0.05 and **P<0.01.

**Table 3:** Thigh muscle strength before and after training.

	CNT (n = 14)			IWT (n = 13)			*P*
	Before	After	*P* (Time)	Before	After	*P* (Time)	(Group×time)
Operated side							
n	17	17		16	16		
F_EXT_ (Nm)	74.6±7.7	74.5±7.5	NS	83.8±5.2	92.5±4.9	NS	NS
(Nm/kg)	1.36±0.15	1.35±0.14	NS	1.56±0.11	1.73±0.12	NS	NS
F_FLX_ (Nm)	36.3±2.3	39.3±2.3*	0.022	37.7±3.3	46.2±2.4**	0.0021	0.036
(Nm/kg)	0.65±0.04	0.70±0.03*	0.028	0.69±0.06	0.85±0.04**	0.0031	0.023
Non-operated side							
n	11	11		10	10		
F_EXT_ (Nm)	96.4±6.5	102.9±11.3	NS	93.3±9.1	88.5±10.1	NS	NS
(Nm/kg)	1.67±0.13	1.72±0.17	NS	1.78±0.19	1.64±0.19	NS	NS
F_FLX_ (Nm)	38.1±4.1	43.4±4.1	NS	40.8±2.1	46.6±2.5**	0.0032	NS
(Nm/kg)	0.64±0.06	0.74±0.07	NS	0.77±0.04	0.87±0.05**	0.0063	NS

Values are given as the means ± SE. F_EXT_, knee extension force; F_FLX_, knee flexion force. Significant differences compared to the value before training, *P<0.05 and **P<0.01.

**Table 4:** Aerobic capacities before and after training.

	CNT (n = 14)			IWT (n = 13)			*P*
	Before	After	*P* (Time)	Before	After	*P* (Time)	(Group×Time)
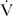 O_2peak_ (ml/kg/min)	16.6±0.8	15.7±0.6	NS	15.8±0.8	17.0±1.1	NS	0.018
HR_rest_^1^ (beats/min)	81±4	81±3	NS	81±2	79±2	NS	NS
HR_peak_ (beats/min)	141±4	143±4	NS	139±4	138±6	NS	NS
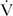 O_2AT_ (ml/kg/min)	13.8±0.6	13.5±0.6	NS	13.7±0.4	15.5±0.5**	0.0021	0.0016
HR_rest_^2^ (beats/min)	77±2	79±2	NS	77±2	78±2	NS	NS
HR_AT_ (beats/min)	108±2	109±2	NS	110±2	114±2*	0.045	NS

Values are given as the mean ± SE. 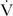
O_2peak_, peak aerobic capacity for walking; HR_rest_^1^, heart rate at rest before starting the exercise for the 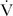
O_2peak_ measurement; HR_peak_, peak heart rate at 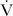
O_2peak_; 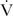
O_2AT_, anaerobic threshold for cycling; HR_rest_^2^, heart rate at rest before starting the exercise for the 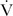
O_2AT_ measurement; and HR_AT_, heart rate at 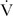
O_2AT_. Significant differences compared to the value before training, *P<0.05 and **P<0.01.

## Results

The adherence to the program was 100% in IWT (including one subject who was excluded from the analysis). Additionally, there were no adverse events, including musculoskeletal injuries and falling, during the training period.

**Table 1** shows the physical characteristics of subjects in the IWT and CNT with no significant differences between groups.

**Table 2** shows the total energy expenditure due to physical activity in both groups. We found that the fast walking time was two-fold higher in IWT than in CNT during and after training periods (both, *P* = 0.001). Additionally, the energy expenditure for fast walking was higher in IWT than in CNT during the training periods (*P* = 0.018 and *P* = 0.047, respectively).

The body mass after training was 57.6±2.6 kg in CNT and 54.1±1.5 kg in IWT and it did not significantly differ from the mass before training in either of the two groups (*P* = 0.20 and *P* = 0.90, respectively).

**Table 3** shows the thigh muscle strength before and after training in both groups. F_EXT_ and F_FLX_ before and after training in both groups tended to be lower on the operated side than on the non-operated side in each group, but there were no significant differences (all, *P*>0.05). We found that F_FLX_ (Nm/kg) increased on the operated and non-operated sides of IWT (*P* = 0.0031, (1-β) = 0.95, and *P* = 0.0063, (1-β) = 0.95, respectively), while it only increased on the operated side of CNT (*P* = 0.028, (1-β) = 0.62) with an interactive effect of group (CNT and IWT)×time (before and after training) for F_FLX_ on the operated side (*P* = 0.023). This result suggests that the increase in F_FLX_ on the operated side was significantly higher in IWT than in CNT.

**Table 4** shows the aerobic capacity before and after training in both groups. 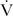
O_2peak_ tended to increase in IWT (*P* = 0.080), while it remained unchanged in CNT (*P* = 0.13), with an interactive effect of (group×time) on the change (*P* = 0.018), suggesting that the increase in IWT was significantly higher than that in CNT. Similarly, 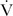
O_2AT_ increased in IWT (*P* = 0.0021, (1-β) = 0.96), while it remained unchanged in CNT (*P* = 0.38) and there was an interactive effect of (group×time) on the change (*P* = 0.0016), suggesting that the increase was significantly higher in IWT than in CNT. The HR_peak_ remained unchanged after training in both groups, but HR_AT_ increased after the training in IWT (*P* = 0.045, (1-β) = 0.53).

**Figure 2** summarizes the changes in the F_EXT_ and F_FLX_ on the operated and non-operated sides after training in both groups and after correcting for the values before training by ANCOVA. Although F_EXT_ on both sides remained unchanged after the training in both groups, F_FLX_ increased by 23% (*P* = 0.0031) and 14% (*P* = 0.0063) on the operated and non-operated sides, respectively, after the training in IWT, while it increased only on the operated side of CNT (*P* = 0.028). The increase on the operated side was significantly higher in IWT than in CNT (ANCOVA, *P* = 0.002, (1-β) = 0.95). The minimum sample size for detecting the difference in the increases in F_FXT_ between group at α = 0.05 and (1-β) = 0.9 was 12 for each group, which is smaller than the size in the present study.

**Figure 3** shows the changes in 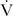
O_2peak_ and 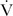
O_2AT_ after training in both groups. Although both remained unchanged in CNT (both, *P*>0.10), the increases in the 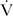
O_2peak_ and 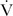
O_2AT_ in IWT were 8% (*P* = 0.08) and 13% (*P* = 0.0021), respectively, which were significantly higher in IWT than in CNT (ANCOVA, *P* = 0.018, (1-β) = 0.68 and *P* = 0.0016, (1-β) = 0.94, respectively). The minimum sample size for detecting the differences in the changes in the 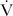
O_2peak_ and 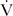
O_2AT_ between groups at α = 0.05 and (1-β) = 0.9 were 23, which is more than in the present study, and 12, which is less than in the present study, for each group, respectively.

The hip pain score was not significantly different between the groups before training (*P*>0.55). After training, the score remained unchanged in both groups on the operated side (*P*>0.06) and on the non-operated side (*P*>0.06).

The walking satisfaction score significantly increased in IWT from 44±9 to 55±8 (*P* = 0.047, (1-β) = 0.53) but not in CNT, whose values changed from 55±10 to 43±10 (*P* = 0.24) with no significant interactive effect of (group×time) (*P* = 0.27).

The “vitality” score in SF-36 significantly increased in IWT from 45±3 to 52±2 (*P* = 0.005, (1-β) = 0.87) but not in CNT, whose values changed from 48±3 to 52±3 (*P* = 0.19). However, there was no significant interactive effect of (group×time) (*P* = 0.24). There were no significant changes in the other items of SF-36 before and after training in either group (*P* = 0.07–1.0).

## Discussion

In the present study, all THA patients in IWT performed fast walking for more than an average of 60 min per week during the 12-wk training period, leading to increased F_FLX_ on the operated side and increased aerobic capacity, which were accompanied by a lack of hip pain.

We compared the 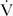
O_2peak_, F_EXT,_ and F_FLX_ before training in CNT and IWT in the present study with those in each of 3 groups that were divided equally according to 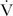
O_2peak_ (n = 156, each) in the previous study; [18] in that study, we examined the effects of 4-month IWT in 468 middle-aged and older women without THA and with an average age of ∼65 years, which was ∼5 years older than in the present study. We found that the 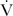
O_2peak_ and F_EXT_ in the present study were not significantly different from those in the lowest 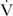
O_2peak_ group (*P*>0.05), while they were significantly lower than those in the middle 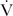
O_2peak_ group (*P*<0.01). In contrast, the F_FLX_ was significantly lower than that in the lowest 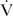
O_2peak_ group (*P*<0.01). Therefore, the 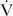
O_2peak_ and F_EXT_ in the subjects in the present study belong to the bottom 30% group of the age-matched healthy population [20] with a significantly lower F_FLX_ than in that group.

As indicated in **Table 2**, the fast walking time was 87 min/wk higher in IWT than in CNT. Nemoto et al. [17] examined the effects of 5-month IWT on 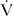
O_2peak_, F_EXT_, and F_FLX_ in 31 middle-aged women and older women with an average age of ∼65 years and no THA, using a target similar to that of the present study, and the researchers reported that the total fast walking time was 32 min/day, the number of walking days was 4.5 days/wk, and the total fast walking time was 144 min/wk. Additionally, Morikawa et al. [18] examined the effects of 4-month IWT on lifestyle-related diseases in 468 middle-aged and older women with an approximate age of ∼65 years lacking THA, and the authors reported that the total fast walking time was 22 min/day, the number of walking days was 3.9 day/wk, and the total fast walking time was 85.8 min/wk. Similarly, Okazaki et al. [29] examined the effects of macronutrient supplementation during 5-month IWT on the thigh muscle strength in 35 middle-aged and older women with an approximate age of ∼61 years and reported that the total fast walking time was 21.5 min/day, the number of walking days was 4.1 days/wk, and the total fast walking time was 88.2 min/wk. Therefore, the IWT subjects likely accomplished the target of more than 60 min/wk of fast walking at an intensity of more than 70% 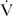
O_2peak_ as in the previous studies [17],[18].

As shown in **Table 3** and **Figure 2**, F_FLX_ in IWT increased significantly by 23% on the operated side and by 14% on the non-operated side, while F_EXT_ remained unchanged after training.

Previous studies have suggested that the response of F_FLX_ to IWT is more sensitive than that of F_EXT_. [17], [18] Nemoto et al. [17] reported that after 5-month IWT, the increase in F_FLX_ was 16% higher than the 12% increase in F_EXT_. Similar results were also reported in previous studies. [17], [27] The precise reasons for the less sensitive response of F_EXT_ remain unclear; however, it might be because the F_FLX_ value is lower than the F_EXT_ value before training. According to the current American College of Sports Medicine guidelines, [30] repetitive contractions above a given percent of maximal force are recommended for increasing muscle strength. In other words, if muscles generate more than a given force in repetitive contractions during training, the strength will increase, but the strength will not otherwise increase. Indeed, Nemoto et al. [17] suggested that the thigh muscle strength remained unchanged after a 5-month moderate-intensity (50% 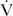
O_2peak_) continuous walking training regimen, while it increased after IWT, which is similar to the results for IWT in the present study. Therefore, the higher sensitivity of F_FLX_ to IWT compared that of F_EXT_ was because the relative exercise intensity was sufficiently high to increase the strength in F_FLX_ but not in F_EXT_.

As shown in **Table 4** and **Figure 3**, the 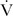
O_2peak_ and 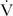
O_2AT_ in IWT increased by 8% and 13%, respectively, while both remained unchanged in CNT.

Nemoto et al. [17] suggested that the 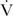
O_2peak_ in individual subjects was significantly correlated with their thigh muscle strength and that the increase after 5-month IWT was accompanied by an increase in thigh muscle strength, suggesting that the changes in 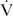
O_2peak_ and 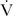
O_2AT_ were caused by the increased oxygen extraction rate in the muscle tissue due to increases in muscle mass, capillary density, and cellular oxidative capacity.

In the present study, the hip pain score remained unchanged in IWT. Generally, THA patients tend to be physically inactive, fearing acetabular cap wear of the implanted joints due to more frequent friction on the surface during walking; [31] however, recent studies have suggested that the wear is not related to the daily physical activity level [32] and, furthermore, the incidence of joint replacement was much higher in patients with less regular exercise, which is most likely due to the reduction in the surrounding muscle mass supporting the joint. [33] We found that the walking satisfaction score, “vitality” in SF-36 in IWT, was improved as the thigh muscle strength increased after training (**Table 3**), which is consistent with the results in the previous study [33].

### Limitations

In the present study, we did not measure the hip muscle strength; however, Judd et al. [33] suggested that the hip flexor and abductor forces recovered to those in the age-matched healthy older women within 12 months after surgery in THA patients, while the hip extensor and knee flexor and extensor forces remained ∼20% lower than in those in healthy women. In the present study, we confirmed that the F_EXT_ and F_FLX_ before training in both groups were ∼20% lower than those in the age-matched total population of healthy women; [18] therefore, the hip and thigh muscle strength likely matched those that were previously reported for THA patients [33].

We only monitored the improvement in daily physical activity for one week after training. Therefore, it is unclear whether the patients would continue IWT afterwards; however, it has been suggested that the adherence rate of 4 months of IWT was 94% in age-matched healthy women, which was due to the instruction service that provided achievement-based feedback over the internet. [18] Therefore, we surmised that most THA patients would continue IWT if the service was provided afterwards.

In the present study, a mild exercise control group was not included; however, Nemoto et al. [17] reported in middle-aged and older people, the F_EXT_ and F_FLX_ significantly increased after high intensity interval walking training for 5 months, while it did not increase after moderate intensity continuous walking training, although the time and steps for training were less in high intensity interval walking training group than in the moderate intensity continuous walking training group. Accordingly, they suggested that high intensity interval walking training increased the thigh muscle strength as resistance training by using machines recommended by the ACSM. [30] We confirmed this in THA patients in the present study.

The subjects in the present study had relatively low BMIs and were middle-aged and older Japanese women. Because this is the first study to evaluate the effects of IWT on THA patients, further studies are needed to assess whether our findings are true for a fatter, Western population.

As mentioned previously, the physical fitness in the subjects in the present study belongs to the bottom 30% group of the age-matched healthy population. [18] Morikawa et al. [18] suggested that the symptoms of life-style related diseases, including hypertension, hyperglycemia, obese, and dyslipidemia, were the worst in the group, but the symptoms improved the most with most increased physical fitness after 5-month interval waling training. Future studies are needed to evaluate the effects of interval walking training on life-style related diseases in THA patients.

### Conclusion

IWT might be an effective home-based training regimen for preventing muscle atrophy due to reduced daily physical activity in THA patients.

## Supporting Information

Check list S1 CONSORT checklist.
(DOC)

Protocol S1 Trial Protocol.
(DOCX)

Protocol S2 Trial Protocol in original language (in Japanese).
(DOC)

Medical Research Ethics Review Application Form S1 Medical Research Ethics Review Application Form.
(DOC)

Medical Research Ethics Review Application Form S2 Medical Research Ethics Review Application Form in original language (in Japanese).
(DOC)
