# Supplementary material for: Effects of Home-Based Interval Walking Training on Thigh Muscle Strength and Aerobic Capacity in Female Total Hip Arthroplasty Patients: A Randomized, Controlled Pilot Study
Source: PLoS One. 2014 Sep 30;9(9):e108690. doi: 10.1371/journal.pone.0108690 (PMC4182539; doi:10.1371/journal.pone.0108690)
Supplement: Protocol S1 — Trial Protocol. (DOCX) [file pone.0108690.s002.docx]

**Effects of home-based interval walking training on thigh muscle strength and aerobic capacity in female total hip arthroplasty patients: A randomized controlled pilot study**

**Protocol**

**Background**

Total hip arthroplasty (THA) is well-accepted surgical procedures in orthopedic with advanced arthritic disorders at hip joint.[1] Early targeted rehabilitation has been shown to reduce hospital length of stay without an increase in complication rates after THA.[2] However, it is well known that THA patients have significant muscular atrophy and weakness in the operated limb that can persist for at least 2 years[3,4] with mobility deficits remaining for a number of years.[1,5–8] Previous study has also indicated that muscle weakness of the operated hip is a major risk associated joint instability, prosthesis loosening, risk of fall, and other complications in THA patients.[9–12] Therefore, continual practice of exercise program is important for THA patients after hospital discharge, not only to prevent declining of muscle strength, but also to maintain a highly functional level and to prevent complications.

　Exercise programs such as muscle strengthening exercise and functional task have been shown to improve muscular strength, walking speed, and mobility in THA patients.[13,14] However, these programs are high cost, because they need to supervision of professional staff at hospital or rehabilitation center. In addition, some THA patients are excluded because difficulties with mobility and transport to a center preclude participation.^15, 16^ On the other hand, some researchers reported effect of home-based exercise program for THA patients without supervision.[15,16] However, these programs showed low adherence rate (50-70%). Therefore, we must develop a convenient and effective exercise program for THA patients.

Recently, we demonstrated that high-intensity interval walking training (IWT) consisting of 5 sets of fast walking above 70% peak aerobic capacity for walking (WVO_2peak_) for 3 min followed by 3 min of slow walking below 40% WVO_2peak_ per day more than 4 days/week for 5 months, increased WVO_2peak_ by ~10%, thigh muscle strength by ~10%.[17,18] In addition, the adherence to the IWT was very high (95%) in these studies. IWT is based on walking, and subjects can practice training at their home without supervision.

**Purpose**

- To examine the effectiveness to the physical function and psychosocial function of IWT on THA patients.
- To examine the safeness of IWT on THA patients.

We hypothesized that THA patients can practice the IWT in safety, and IWT will be improved physical function and psychosocial function in THA patients.

**Methods**

**Participants:**

Participants of this study will be recruited from the Department of Orthopedics, Hamamatsu University School of Medicine, University Hospital and patient advocacy group in Hamamatsu city. The inclusion criteria of participants were having undergone the primary THA before this study, and being able to walk independently. We do not consider using any assistive device. The exclusion criteria consisted of acetabular and/or femoral prosthesis failure and comorbidity such as cardiopulmonary, neurologic, or cognitive diseases. Participants will be randomly divided into control group or interval walking group. The randomization will be done by the one researcher, using a permuted block randomization (4 of block sizes).

**Tests:**

All assessments for subjects at the Hamamatsu University School of Medicine, University Hospital will be done at the department of rehabilitation. The assessments will be done before starting training (baseline), after 12 weeks (end of training),.

Isometric bilateral knee extension and flexion forces will be measured with an isometric dynamometer (GT-330; OG Giken, Tokyo, Japan). The higher value of 2 trials of extension and flexion forces, respectively.

WVO_2peak_ will be measured by previous reported methods using by tri-axial accelerometer (JD-Mate; Kissei Comtec, Matsumoto, Japan). [17,18]Subjects with JD-Mate on their back walked for 3 minutes on a flat floor at 3 graded subjective velocities: slow, moderate, and fast at ~25°C room temperature and ~60% relative humidity. At the same time, heart rate was counted with an electrocardiogram and 3-dimensional acceleration was measured at 10-millisecond intervals and recorded with 5-second memories as averaged values.[19,20] The total impulse from the accelerometer was transferred to a computer and converted to the oxygen consumption rate (VO_2_) using a previously reported equation.[19,20] WVO_2peak_ and peak heart rate (HR_peak_) for walking values are those for the last 30 seconds at maximal walking speed.

We will determine CVO_2AT_ at ~25°C room temperature and ~60% relative humidity with an expired gas analysis system (AE-310S; Minato, Tokyo, Japan). VO_2_ and carbon dioxide production (VCO_2_) were measured every breath. Exercise intensity was increased by 10 watts per minute and stopped when the anaerobic threshold was judged to have been reached according to the standard method,[21–23] by viewing the VO_2_ vs. VCO_2_ relationship displayed on the screen of the system during exercise. We also determined heart rate at CVO_2AT_ (HR_AT_).

Total energy expenditure for physical activity will be determined by summing the VO_2_ (O_2_ ml/kg/min) before (1 wk), during (12 wks), and after (1 wk) training periods, respectively, after excluding energy expenditure at rest, assuming that it was constant during these periods, and is presented as O_2_ ml/kg/wk. Walking steps will be determined by summing steps for the respective periods and are presented as steps/wk.

Hip joint pain will be evaluated with a scale of 100mm VAS, where 0 represents “no pain” and 100 represents “worst pain”.

Walking satisfaction will be evaluated using 0 as “no satisfaction” and 100 as “full satisfaction”.

The SF-36^®^ will be used to evaluate quality of daily life.[24,25]

**Intervention**

**Control group:**

Subjects in CNT will be instructed to maintain the same lifestyle as before training. Every 2 weeks during the training period for IWT, they will be visited the hospital, and energy expenditure by daily physical activity and steps during the day, except for sleeping and bathing periods, will be transferred from the tracking devices to the central server in the administrative center over the internet but with no automatic analysis and reporting, unlike in IWT.

**Interval walking group:**

Subjects in IWT will be visited a gym near the hospital and received instructions about the exercise program. They will be told to repeat 5 or more sets of 2- to 3-minute low-intensity walking intervals at ~40% of the pre-training WVO_2peak_, followed by a 3-minute interval of high-intensity walking at >70% but <85% WVO_2peak_, >4 days/wk, so that total fast walking time per week reached >60 min. The intensity and steps were monitored with JD-Mate, worn on the mid-clavicular line of the right or left waist. A beeping signal alerted subjects when a change of intensity was scheduled, and another sound told them when their walking intensity had reached the target level every minute. Once subjects had learned the program, they were allowed to choose their training time each day. In addition, in IWT, energy expenditure by physical activity other than IWT and steps was measured with JD-Mate during the day, except for bathing and sleeping periods, while the beeping signal and sounds of the device were switched off.

Every 2 weeks, subjects will be visited the hospital, and data from the tracking devices will be transferred to a central server in the administrative center over the internet for automatic analysis and reporting based on the database on the effects of IWT in more than >3,000 middle-aged and older people with no THA, which we call the e-Health Promotion System (Kissei Comtec).[26] Physical therapists will use these reports to track daily walking intensity and other parameters given in **Table 2** to instruct subjects on how best to achieve the target levels. If the targets will be not met, they will encourage subjects to increase their efforts to achieve them.

**References**

1. Nilsdotter AK, Roos EM, Westerlund JP, Roos HP, Lohmander LS (2001) Comparative responsiveness of measures of pain and function after total hip replacement. Arthritis Rheum 45: 258–262.

2. Iyengar KP, Nadkarni JB, Ivanovic N, Mahale A (2007) Targeted early rehabilitation at home after total hip and knee joint replacement: Does it work?

3. Rasch A, Byström AH, Dalén N, Martinez-Carranza N, Berg HE (2009) Persisting muscle atrophy two years after replacement of the hip. J Bone Joint Surg Br 91: 583–588.

4. Rasch A, Dalén N, Berg HE (2010) Muscle strength, gait, and balance in 20 patients with hip osteoarthritis followed for 2 years after THA. Acta Orthop 81: 183–188.

5. Murray MP, Gore DR, Brewer BJ, Gardner GM, Sepic SB (1979) A comparison of the funtional performance of patients with Charnley and Müller total hip replacement. A two-year follow-up of eighty-nine cases. Acta Orthop Scand 50: 563–569.

6. Victor CR (1987) Rehabilitation after hip replacement: a one year follow up. Int J Rehabil Res 10: 162–167.

7. Sicard-Rosenbaum L, Light KE, Behrman AL (2002) Gait, lower extremity strength, and self-assessed mobility after hip arthroplasty. J Gerontol A Biol Sci Med Sci 57: M47–51.

8. Madsen MS, Ritter MA, Morris HH, Meding JB, Berend ME, et al. (2004) The effect of total hip arthroplasty surgical approach on gait. J Orthop Res 22: 44–50.

9. Long WT, Dorr LD, Healy B, Perry J (1993) Functional recovery of noncemented total hip arthroplasty. Clin Orthop Relat Res: 73–77.

10. Perrin T, Dorr LD, Perry J, Gronley J, Hull DB (1985) Functional evaluation of total hip arthroplasty with five- to ten-year follow-up evaluation. Clin Orthop Relat Res: 252–260.

11. Dorr LD, Wan Z (1998) Causes of and treatment protocol for instability of total hip replacement. Clin Orthop Relat Res: 144–151.

12. Lachiewicz PF, Soileau ES (2002) Stability of total hip arthroplasty in patients 75 years or older. Clin Orthop Relat Res: 65–69.

13. Mossey JM, Mutran E, Knott K, Craik R (1989) Determinants of recovery 12 months after hip fracture: the importance of psychosocial factors. Am J Public Health 79: 279–286.

14. Magaziner J, Simonsick EM, Kashner TM, Hebel JR, Kenzora JE (1990) Predictors of functional recovery one year following hospital discharge for hip fracture: a prospective study. J Gerontol 45: M101–7.

15. Sashika H, Matsuba Y, Watanabe Y (1996) Home program of physical therapy: effect on disabilities of patients with total hip arthroplasty. Arch Phys Med Rehabil 77: 273–277.

16. Jan M-H, Hung J-Y, Lin JC-H, Wang S-F, Liu T-K, et al. (2004) Effects of a home program on strength, walking speed, and function after total hip replacement. Arch Phys Med Rehabil 85: 1943–1951.

17. Nemoto K, Gen-no H, Masuki S, Okazaki K, Nose H (2007) Effects of high-intensity interval walking training on physical fitness and blood pressure in middle-aged and older people. Mayo Clin Proc Mayo Clin 82: 803–811.

18. Morikawa M, Okazaki K, Masuki S, Kamijo Y, Yamazaki T, et al. (2011) Physical fitness and indices of lifestyle-related diseases before and after interval walking training in middle-aged and older males and females. Br J Sports Med 45: 216–224.

19. Iwashita S, Takeno Y, Okazaki K, Itoh J, Kamijo Y, et al. (2003) Triaxial accelerometry to evaluate walking efficiency in older subjects. Med Sci Sports Exerc 35: 1766–1772.

20. Yamazaki T, Gen-No H, Kamijo Y-I, Okazaki K, Masuki S, et al. (2009) A new device to estimate VO2 during incline walking by accelerometry and barometry. Med Sci Sports Exerc 41: 2213–2219.

21. Wasserman K, Whipp BJ, Koyl SN, Beaver WL (1973) Anaerobic threshold and respiratory gas exchange during exercise. J Appl Physiol 35: 236–243.

22. Davis JA, Frank MH, Whipp BJ, Wasserman K (1979) Anaerobic threshold alterations caused by endurance training in middle-aged men. J Appl Physiol 46: 1039–1046.

23. Beaver WL, Wasserman K, Whipp BJ (1986) A new method for detecting anaerobic threshold by gas exchange. J Appl Physiol 60: 2020–2027.

24. Fukuhara S, Ware J, Kosinski M (1998) Psychometric and clinical tests of validity of the Japanese SF-36 Health Survey. J Clin Epidemiol 51: 1045–1053.

25. Fukuhara S, Bito S, Green J, Hsiao a, Kurokawa K (1998) Translation, adaptation, and validation of the SF-36 Health Survey for use in Japan. J Clin Epidemiol 51: 1037–1044.

26. Nose H, Morikawa M, Yamazaki T, Nemoto K-I, Okazaki K, et al. (2009) Beyond epidemiology: field studies and the physiology laboratory as the whole world. J Physiol 587: 5569–5575.
