# Supplementary material for: Effects of Home-Based Interval Walking Training on Thigh Muscle Strength and Aerobic Capacity in Female Total Hip Arthroplasty Patients: A Randomized, Controlled Pilot Study
Source: PLoS One. 2014 Sep 30;9(9):e108690. doi: 10.1371/journal.pone.0108690 (PMC4182539; doi:10.1371/journal.pone.0108690)
Supplement: Protocol S2 — Trial Protocol in original language (in Japanese). (DOC) [file pone.0108690.s003.doc]

臨床試験実施計画書

1. 研究の背景・意義

変形性関節症は、高齢者の要支援・要介護の原因として主要な位置を占める病態のひとつである。なかでも変形性股関節症（以下股OA）は、有病率は60歳以上の女性で2％を占め、実際のリハビリテーションの現場で、股OAに対する骨切り術や人工股関節置換術を受けた患者の診療にあたる機会は非常に多い。

我が国では、股OAに対する人工関節置換術は主に疼痛除去のために導入され、2004年には，年間32,000件を超える人工股関節置換術（以下THA）が行われた。しかし，包括医療制度の導入等により入院期間は約3～4週間に短縮されるようになった結果、関節可動域や筋力等において十分な機能回復に至らないまま、自宅での生活を再開することも多くなった。実際に、THA施行1年後の追跡調査では、術側の下肢筋力は非術側の水準まで到達してなかったり、また、姿勢安定性についても非術側と比較して術側で有意に低下していることが報告されている。

THA患者は、術前後で比較すると、術後は歩容が改善することで積極的になる人を日常診療中、よく経験する。松本らは、THA患者について歩容の主観的評価と健康関連QOL尺度であるSF-36の関連をみると、歩容に満足している人ほど自分の身体機能に満足し、精神的に安定した生活を送っている事を報告している。従って、THA患者においては、下肢筋力の改善は歩容を改善し、健康関連QOLも改善することが予測される。

THA患者に対するリハビリテーションは、前述の通り在院日数の短縮化や術創部の疼痛のため、筋力強化や歩行などのトレーニングが十分実施できず、人工関節の脱臼予防等のセルフケア方法の指導が中心となる傾向がある。

また退院後外来通院リハビリに切り替える際も、通院手段等の社会背景的問題や、医療保険上のリハビリテーション日数の制限等により、通院困難な例が少なくない。そこで、在宅での運動プログラムが重要となってくる。Jacksonらも術後4～12ヶ月のTHA患者に対し、在宅で監視下による筋力トレーニング、姿勢安定性トレーニングを実施した結果、24～41.2％の下肢筋力の向上を得たと報告している。しかし、現状では在宅における運動指導者は圧倒的に不足している。こうした現状を踏まえると、患者自身で実施可能な簡便でかつ効果的なトレーニング方法の開発が急務と考えられる。

ところで、松本市をはじめとした多くの自治体で、健常な中高年を対象として、3分間ごとに最大歩行速度の70％以上の速歩と30％の緩歩を繰り返す、「インターバル速歩トレーニング」が実施されている。このトレーニングは、携帯型運動量計測器（熟大メイト、キッセイコムテック（株）社製）を用いて、各個人の機能に応じた個別運動処方が可能となっており、生活習慣病予防、体力増強プログラムとしての有効性が明らかになっている。

我々は、この「インターバル速歩トレーニング」が、THA患者の在宅トレーニングに適しているのでないかと考えた。しかしながら、THA患者に対するこのトレーニング法」の運動機能や健康増進効果は明らかでない。

1. 研究の目的

- THA患者の個人主体の在宅トレーニングとしてのインターバル速歩トレーニングの有効性を判定する。
- THA患者の個人主体の在宅トレーニングとしてのインターバル速歩トレーニングの安全性を判定する。

1. 薬剤情報

該当なし

1. 研究対象

（１）選択方針

　試料などの提供者に対する医学的・精神的影響及びそれらに配慮した研究方法の是非等について慎重に検討する。特にインフォームド・コンセントの手続及び方法、個人情報の保護の方法、研究により予測される結果及びその開示の考え方、試料等の保存及び使用の方法並びに遺伝カウンセリングの考え方について明確に説明する。また、試料などの提供者が、疾病や薬剤反応性異常を有する場合及びそれらの可能性のある場合には、該当する病名又はそれに相当する状態像を告知する。その上で、該当する患者に当研究に関して患者用説明文書を用いて十分に説明し、インフォームド・コンセントが得られれば、研究を開始する。

（２）選択基準

- 医療機関によるリハビリテーションを受けていないTHA後成人男女
- 自立歩行が可能である者（歩行補助具の使用は可）

(３) 除外基準

・THAデバイスに障害のある者

・循環器疾患、呼吸器疾患、認知機能障害の既往を持つ者

1. 研究の方法・治療計画

（１）デザイン（スケジュール）

上記の選択基準に合致し、トレーニング開始前検査などにて試験参加が適切であると判断された症例に対し、インターバル速歩トレーニングによる介入を開始する。

【対象の割付】

対象は、IWT群及びCNT群の2群に無作為に割り付ける。無作為化は置換ブロック法を使用し（ブロックサイズは4）、1名の研究者のみで実施する。

【研究スケジュール】

|  | 期間 |  |  | |
| --- | --- | --- | --- | --- |
| IWT群 | **トレーニング^※^** |  |  | |
| CNT群 | 普段通りの生活 |  |  | |
| 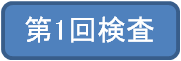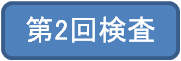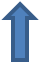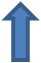   |  | | --- | |  |  | |  |
|  |  |  | |  |
|  |  |  | |  |

【^※^インターバル速歩トレーニング】

最大歩行速度の70％以上の速歩3分間と30％以下の緩歩3分間を1セットとする。これを１日に5セット行い、週に4日以上実施するように指導する。

この際、歩行速度は携帯型運動量計測器（熟大メイト）^※^（特許3571272）を使用し、これにより歩行速度は管理する。

※熟大メイトは、3軸型加速度計が内蔵されており、設定された速歩の速度に達すると通知音が鳴り、3分間経過すると再度通知音が鳴り、緩歩の合図を出す。設定した緩歩速度に達して3分間経過すると、再度速歩へ切り替える通知音が鳴り、上記を繰り返す。

歩行強度は、測定時にNemoto（Mayo Clin Proc. 2007;82(7):803-811）らにより報告されている、3段階ステップアップ法に従って、測定及び処方する。

（２）観察、検査、評価項目、およびこれらに関する方法と時期

【評価項目】

1. 形態測定

・身長

・体重、体脂肪率　 ：体脂肪計付きヘルスメーターを使用し、裸足で測定する。

・BMI　　　　　　 ：身長と体重から算出する。

1. 筋力測定

・下肢筋力：筋力測定装置（アイソフォースGT-330、オージー技研社製）を使用し、下記の運動について測定する。検者は同一とする。

膝関節　：　屈曲・伸展

1. 運動耐容能（最大酸素摂取量）

・3軸加速度計を使用し（熟大メイト、キッセイコムテック）、Nemoto（Mayo Clin Proc. 2007;82(7):803-811）らにより報告されている、3段階ステップアップ法に従って、測定する。同時に心拍数を測定する。3軸加速度は0.1秒毎に、心拍数は5秒毎に平均化し記録する。最大酸素摂取量および最大心拍数はは、最大歩行速度における最後の30秒間の値を代表値とする。

1. 運動耐用能（無酸素性作業閾値）

・呼吸代謝測定装置（エアロモニタAE-310、ミナト医科学社製）を使用し、自転車エルゴメーターによるランプ負荷による、最大下運動負荷試験を実施する。無酸素性作業閾値はV-slope法を使用し測定する。また、無酸素性作業閾値における心拍数を記録する。

1. 身体活動量

- 3軸加速度計を使用し（熟大メイト、キッセイコムテック）を使用し測定する（酸素摂取量；O_2_ ml/kg/分）。数値は、週ごとの値で平均化する(O_2_ ml/kg/週)。測定は、トレーニング期間前（1週）、トレーニング期間（12週）、トレーニング期間後（1週）とする。
- 歩数も同様に測定する。歩数は週ごとの値に平均化する（歩/週）。

1. 股関節痛

- VASを使用する。
- 0は「全く痛くない」、100を「最大の痛み」とする。

1. 歩行満足度

- VASを使用する。
- 0は「全く満足していない」、100を「最高に満足している」とする。

1. 健康関連QOL

・SF‐36を使用する。

【測定時期】

合計2回の測定時期を設ける。測定項目については上記のものとし、2回全てで内容は同一とする。

・第1回測定：試験開始前

・第2回測定：試験開始12週間後

1. 有害事象の評価

＜参考：有害事象とは、本研究に参加した被験者に生じたあらゆる好ましくない、あるいは意図しない徴候（臨床検査値の異常を含む）、症状または病気のことをいい、投与された医薬品や治療処置との因果関係を問わない。＞

　（１）有害事象・有害反応の評価

本研究中に有害事象が生じた場合、最前の処置を行い、経過を観察するとともに、その内容、程度、発現時期、処置、転帰、本治療との因果関係を詳細に調査する。本研究で実施する運動トレーニングとの因果関係がなしと判定した場合はその根拠を症例報告書に書く。

（２）有害事象の報告

責任医師は必要と認めた場合、他の試験担当医師に対して、当該有害事象の内容について文面で報告する。

（３）有害事象発現時の被験者のフォローアップ

試験担当医師は、有害事象が認められた場合は最善の処置・治療を行い、副作用については改善あるいは安定するまで可能な限り観察を継続する。

（４）予期される有害事象・有害反応とその発現率

　　　・運動トレーニングによる筋肉痛、関節痛

　　　・転倒による捻挫、骨折

７．研究に参加することにより期待される利益および、起こりうる危険ならび

　　に必然的に伴う心身に対する不快な状態

【利益】

IWT群は12週間のインターバル速歩トレーニング直後で下肢筋力、体重、体脂肪率、ＢＭＩ、歩行満足度、健康関連QOLが改善することが考えられる。また、IWT程度の運動では股関節痛は変化がないことが予想される。

】起こりうる危険】

・運動トレーニングによる筋肉痛、関節痛

　　　・転倒による捻挫、骨折

８．試験中止基準

（１）症状が悪化し、研究を継続することが困難、もしくは別の治療に変更する必要があると医師が判断した場合

1. 合併症の悪化もしくは有害事象の発現により、医師が研究を中止すべきと判断した場合
2. この臨床研究への参加が不適切であることが判明した場合
3. 患者の都合により必要な観察・検査の実施が不可能であることが判明した場合
4. 患者が本臨床試験実施計画に著しく違反し、この臨床研究の適正な評価が不可能であると医師が判断した場合

（６）患者が同意を撤回した場合

（７）この臨床研究全体の中断もしくは中止が決定した場合

９．症例登録とデータ収集

（１）症例登録

担当医師は、本研究の症例選択基準を見たし、除外基準に該当しないことが確認された症例に対し、本研究に参加に関する同意を文書にて取得し、研究代表者に連絡し、症例登録を行う。

1. データの集積

登録症例のデータは、調査票を用いて浜松医科大学医学部附属病院リハビリテーション部に集積する。

１０．統計学的事項

（１）解析方法

各群の評価指標を、それぞれトレーニング前後の値を比較する。

また、各測定時期における、群間の差についても比較検討する。

（２）予定登録症例数

　　　30症例（IWT群15症例、CNT群15症例）

（３）登録期間

平成21年7月～平成21年8月

（４）追跡期間

平成21年9月～平成21年12月

１１．倫理

1. 患者の保護

本研究に関係するすべての研究者はヘルシンキ宣言および厚生労働省「臨床研究に関する倫理指針」(http://imcj.go.jp/rinri/index.html)を遵守して、本研究を実施する。

　　　　（１）症例の集積および解析に際して匿名化をする。

（２）調査票の報告などには個人名を特定できないようにする。

（３）本研究が公表される場合も被験者の秘密を保全する。

　　（イ）個人情報を含む情報の保護についての具体的方法

本研究にかかわるものは、参加するすべての患者の個人情報を保護するため、以下の事項に配慮する。また、業務上患者の個人情報を知りうるものはその秘匿を保持する。

（１）試料は連結可能匿名化にする。結果の解析を始める前に資料から住所や氏名など個人を特定できる情報を削り、符号を付けて匿名化する。匿名化した符号と患者の個人情報とを連結させる対応表は、個人情報分担管理者が厳重に保管する。また、匿名化コードで整理され個人を特定できる情報を含まない研究結果、資料や診療記録、個人情報も対応表とは別に管理する。個人情報を処理するコンピュータは他のコンピュータと切り離されたものを利用し、コンピュータの外部記憶装置に保管して独立した鍵のかかる場所で厳重に管理する。

（２）すべての試料は研究終了後に直ちに廃棄され、診療記録もそれ以降は本研究のためには使用しない。試料を廃棄する場合には、匿名のまま、密封容器に廃棄あるいは焼却処分する。個人情報、研究記録なども匿名のままシュレッダー処分あるいは電子的に消去する。

　　（ウ）情報の開示

（１）提供者本人が情報の開示を希望する場合は、原則的に結果を開示する。

1. 提供者本人が情報の開示を希望していない場合は、開示しない。
2. 提供者以外が情報の開示を希望する場合は、原則的に結果を開示しない。

１２.　インフォームドコンセント

（ア）被験者への説明

　研究参加に先立って、担当医は施設の倫理委員会承認が得られた同意説明文書を被験者本人（あるいは代理人）に渡して、以下の内容を口頭で詳しく説明する。

　　 □　試料等の提供は任意であり、提供に同意しなくても不利益な対応を受けることはない。

　　　　　 また、いつでも不利益を受けることなく同意を文書により撤回できること

　　　 □　同意が撤回された場合には、当該撤回に係る試料等及び研究結果が連結不可能匿名化されている場合等を除き、廃棄されること

　　　 □　提供者として選ばれた理由

　　　 □　研究の意義、目的及び方法（対象とする疾患、分析方法等。将来の追加、変更が予想される場合はその旨。単一遺伝子疾患等の場合には研究の必要性、不利益を防止するための措置等の特記事項等｡)

　　　 □　予測される研究結果及び提供者等に対して予測される危険や不利益（社会的な差別等社会生活上の不利益を含む。）

　　　 □　提供を受けた試料等又はそれから得られた遺伝情報についての連結可能匿名化又は連結不可能匿名化の別、及び匿名化の具体的方法。匿名化できない場合にあっては、その旨及び理由

　　　 □　提供者及び代諾者等の希望により、研究計画及び研究方法についての資料を入手又は閲覧することができる。その場合、他の提供者等の個人情報の保護や研究の独創性の確保に支障が生じない範囲内であることが遵守されていること。

　　　 □　試料等又はそれから得られた遺伝情報を他の機関へ提供する可能性の有無。提供する場合は、倫理審査委員会により、個人情報の取扱い、提供先の機関名、提供先における利用目的が妥当であることについて、審査されていること

　　　 □　研究の一部を委託する場合、試料等の匿名化の方法等

□ 遺伝情報の開示に関する事項

□ 研究で得られた情報の開示について

□　将来、研究の成果が特許権等の知的財産権を生み出す可能性があること。特許権等の知的財産権を生み出した場合の想定される帰属先（該当する研究のみ）

　　　 □　試料等から得られた遺伝情報は、匿名化された上、学会等に公表され得ること

　　　 □　試料等の保存及び使用方法

　　　 □　研究終了後の試料等の保存、使用又は廃棄の方法。他の研究への利用の可能性の有無と予測される研究内容を含む。

　　　 □　遺伝カウンセリングの利用に係る情報。単一遺伝子疾患等の場合には、遺伝カウンセリングが利用可能であること等（該当する研究のみ）

　　　 □　研究資金の調達方法について

□ 試料等の提供についての補償

　　　 □　研究責任者の氏名及び職名、問い合わせ、苦情等の窓口の連絡先等に関する情報

　　　 □　その他の特記事項

　　　　　　　具体的な特記事項を記入　：特記すべきものなし

（イ）被験者（あるいは代理人）の同意

（１）研究についての説明をおこなった後、被験者が試験の内容をよく理解したことを確認した上で研究への参加を依頼する。すなわち、臨床研究に参加は自由意思にもとづくこと、臨床研究に参加しない場合でも不利益を対応を受けないこと、いつでも、どのような理由でも、何ら不利益を受けることなくこの臨床研究への参加を撤回できることなどを、十分に説明する。

（２）被験者あるいは代理人が研究参加に同意した場合、所定の同意書を用い、医師の説明を受けたのち、同意文書に自署する。

　　　（３）同意取得時期

　　　　　　同意の取得は研究参加の前とする。

１３．プロトコールの遵守

　　　本臨床試験に参加する研究者は、患者の安全と人権を損なわない限りに

おいて本プロトコールを遵守する。

１４．倫理委員会の承認

　臨床試験の計画内容については、人権と安全性について最大限の考慮

をし、かつ科学性および倫理性が審議された上で、浜松医科大学医の倫

理委員会で承認をうける。

１５．記録の保存

　所定の同意書に、説明をした医師、説明を受け同意した患者（あるいは代理人）が、同意を得た日付などを各々自署する。

　同意文書は複写を２部作成し、１部は患者本人に手渡し、１部は施設で保管する。原本はカルテに保管する。診療記録及び測定により作成した評価表は鍵のかかる場所で厳重に管理する。

１６．研究成果の発表

　　　公表の際には、被験者のプライバシーを保全する。

１７．利害の衝突（conflict of interest）と研究資金

　　　＜参考：利害の衝突（conflict of interest）とは、研究成果に影響するような利害関係を指し、金銭及び個人の関係を含む。＞

本研究の計画・実施あるいは報告において、試験の結果及び結果の解

　　　釈に影響を及ぼすような「起こり得る利益の衝突」はない。また、研究　　　　の実施が被験者の権利・利益を損ねることはない。

１８．臨床研究の費用

　　　血液検査は教室の研究費で支払い、無償とする。その他測定についても費用の発生しないものであるため、無償である。

１９．金銭の支払い

研究参加に対する金銭的補償（謝礼金など）はない。

２０．健康被害補償

本臨床研究の実施に起因して被験者に健康被害が生じた場合には、研究担当医師は十分な治療並びにその他の適切な処置を行うとともに、その原因の究明につとめる。

２１．研究組織と責任者

試験責任医師：リハビリテーション科　美津島　隆

試験分担医師：リハビリテーション科　山内　克哉

問合わせ先 ：浜松医科大学

緊急連絡先 ：電話　053-435-2746（リハビリテーション部）（平日8：30〜17：15）

FAX 　053-435-2747
