# Supplementary material for: Effects of Home-Based Interval Walking Training on Thigh Muscle Strength and Aerobic Capacity in Female Total Hip Arthroplasty Patients: A Randomized, Controlled Pilot Study
Source: PLoS One. 2014 Sep 30;9(9):e108690. doi: 10.1371/journal.pone.0108690 (PMC4182539; doi:10.1371/journal.pone.0108690)
Supplement: Medical Research Ethics Review Application Form S1 — Medical Research Ethics Review Application Form. (DOC) [file pone.0108690.s004.doc]

Medical Research Ethics Review Application Form

　July 14, 2009

To the Head of Hamamatsu University School of Medicine

　　　　　　　　　　　　　　　　　　　　　　　Lead researcher, Affiliation: Department of Rehabilitation

　　　　　　　　　　　　　　　　　　　　　　　　　Job title and name: Associate professor, Takashi Mitsushima (seal)

　　　　　　　　　Head of affiliated institution, Job title and name: Associate professor, Takashi Mitsushima (seal)

※Receipt number

| 1.　Study title | | | The development of new home training methods for patients following total hip arthroplasty | |
| --- | --- | --- | --- | --- |
| 2.　Lead researcher | | | Name: Takashi Mitsushima　　Affiliation: Department of Rehabilitation　　　Job title: Associate professor | |
| 3.　Study structure | | |  | |
|  |  | 3-1  On campus | Affiliations, job titles, and names of the lead researcher and research staff | Role |
|  |  |  | Department of Rehabilitation  Associate professor Takashi Mitsushima  Department of Rehabilitation  Assistant professor Katsuya Yamauchi  Department of Rehabilitation  Physical therapist Yutaka Morishima | Research supervision  Medical supervision, measurements, recording, and data analysis  Measurements and recording, teaching intervention methods |
|  | Outside joint research institutions and organizations | 3-2  If the university’s code of ethics and ethical review board decisions are followed | Affiliations, job titles, and names of outside researchers | Role |
|  |  |  | Shinshu University Graduate School of Medicine  Professor Hiroshi Nose | Teaching intervention methods |
|  |  | 3-3  If an independent ethical review board is established | Names of outside joint research institutions and organizations and persons in charge of the study | Role |
|  |  |  |  |  |
| 4. Manager of personal information for this study  (The manager of personal information cannot serve as chief researcher or research staff) | | | Affiliation　　Department of Rehabilitation  Job title　　　Medical staff  Name　　 Hiroshi Irisawa | |
| 5. Purpose of the study | | | To apply “interval walking training,” which has been reported as effective for healthy elderly individuals in The Matsumoto Physical Training Program for Senior Citizens, to at-home training for patients who have undergone total hip arthroplasty, and to investigate the effectiveness of this training in health promotion and improving motor function. | |
| 6. Overview of the study | | | Overview:  Post-total hip arthroplasty patients will be randomly divided into two groups (an interval walking group and control group). Patients in the interval walking group will be instructed to perform the interval walking training where fast walking at 70% of maximum walking speed and slow walking at 30% of maximum walking speed are repeated. Patients in the control group will be instructed to carry on with their lives as usual. The intervention period will be 12 weeks and the effect of training will be investigated in terms of muscle strength, exercise tolerance (maximal oxygen uptake, anaerobic threshold), and amount of physical activity. The effect of training will also be investigated in terms of psychosocial functioning such as walking satisfaction and health-related quality of life (QOL). Changes in hip pain after training will also be examined. | |
|  |  |  | Study subjects (research collaborators): 30 patients who have undergone total hip arthroplasty  Implementation site: Hamamatsu University Hospital Department of Rehabilitation  Predicted sample size: 30 patients  Types and amounts of data collected per patient:  ※Clauses for types and amounts of data shall be added as necessary. Data include treatment information (clinical assessment lists etc.).  Type　Blood　　　　 　　　Amount　　10 ml  Type　Clinical assessment list　Amount　　2 sheets | |
| 7. Study period  　(The limit is 3 years. If the 3 years are exceeded, a continuation shall be applied for. However, the limit for human genome and genetic analysis research is 5 years.) | | | From July 2009 through July 2011 | |
| 8. Approval for joint research institutions in the case of joint research  (1) Approval of the study plan in question  Joint research institutions shall follow all methods and procedures approved by the ethical review board.    (2) Informed consent    Written informed consent shall be obtained from professor Nose of Shinshu University after the lead researcher has fully explained the purpose and content of this study both verbally and in writing.  (3) Anonymization  Yes 　　Samples will be anonymized. Before results are analyzed, personally identifiable information such as addresses and names shall be removed and samples shall be anonymized with reference numbers. The personal information manager shall privately maintain a correspondence table linking the anonymized reference numbers with patient personal information. In addition, data, treatment records, and personal information shall be kept in separate, secure locations.  <Note: Anonymization of data or genetic information shall not be performed if this is acknowledged in the study plan that has been approved by the head of the research institution, the subject or their legal representative consents to this, and the ethical review board has given approval.>  (4) Other special remarks  (5) Countermeasures and outlines of methods and standards of foreign countries and Japan stated in the guidelines in the case of joint research with a country overseas | | | | |
| ☆9. Approach to the disclosure and publication of results obtained in this study | | | | |
| 10. Selection policy for data providers/subjects/research collaborators (only data provided by other research institutions shall be excluded)  (1) Does the subject have a disease or abnormal reaction to drugs, or the possibility of this?  　　　　■　Yes  　　The disease name or corresponding condition  　　　　　Post-total hip arthroplasty  □　No    (2) Will the rationality, validity, and fairness of selection be ensured? (Please describe the specific method used to ascertain rational selection)  　　　　 The medical and psychological effects on subjects and the pros and cons of research methods taking these effects into account will be carefully considered. Methods and procedures for informed consent, methods of protecting personal information, views on the expected results of this study and their disclosure, storage and usage of data, and views on genetic counseling in particular shall be clearly explained. Subjects shall notify us of the disease name or corresponding condition if they have a disease associated with total hip arthroplasty. The patients in question shall be fully briefed on this study using briefing documents and shall participate in this study only if they give their written informed consent. | | | | |
| ＃☆11. Anticipated advantages or risks and disadvantages (including social discrimination) to data providers/subjects and families and considerations regarding methods of preventing these  　(Including study contents related to the need for this research, the medical and psychological effect on the subjects, and the pros and cons of research methods taking these into account in the case that the data provider/subject has a monogenetic disorder for which no treatment or method of prevention has been established in addition to a psychiatric or intellectual disorder.)  (1) Is the research method being employed an already established method?    As described below, the research technique being employed is an already established technique.  Blood sampling will be performed from the vein and carry almost no risk.  Muscle strength measurements will be non-invasive, easy to perform, and carry almost no risk.  Measurements of exercise tolerance will be conducted under strict monitoring by a physician and therefore carry almost no risk.  (2) Are there any anticipated advantages or risks and disadvantages to individuals concerned and their families?  During intergroup comparisons, the advantages or risks and disadvantages of each group shall be compared against those of other groups and recorded in detail. Documents and literature proving this shall be attached.  Advantages: Improved muscle strength and exercise tolerance are expected in terms of physical functioning and improved walking satisfaction and health-related QOL are expected in terms of mental functioning.  Disadvantages: Muscle and joint pain caused by exercise training or fractures and sprains caused by falls.  (3) Responses to any adverse events  The appropriate treatment shall be administered in the case of adverse events. If any serious adverse events occur, these shall be immediately reported to the hospital director.  (4) Will this research impact subjects’ natural surroundings and will safety be ensured?  All data shall be processed and discarded in an appropriate manner, thereby having no impact on subjects’ natural surroundings and ensuring safety. | | | | |
| ＊12. Anticipated risks and disadvantages (including social discrimination) to research collaborators and their families and protection of human rights  (1) Are there any predicted risks or disadvantages to the research collaborators concerned and their families?  (2) The rights of research collaborators concerned and their families not to be subjected to risks or disadvantages  (3) The right to self-determination of the research collaborators concerned and their families | | | | |
| 13. Methods of protecting personal information in the study in question  The following measures shall be taken to safely manage and prevent the disclosure, loss, or damage of personal information. As an organizational safety control measure, the responsibilities and authority of researchers with respect to safety management shall be clearly defined, strict safety management shall be performed, and the implementation of safety management shall be confirmed. As a human security control measure, the signing of non-disclosure agreements with regard to personal information considered sensitive by researchers etc. shall be confirmed. As a physical security control measure, entrance to and exit from the building shall be monitored to prevent the theft of personal information. As a technical security control measure, access to personal information and systems handling this information shall be controlled, countermeasures shall be implemented against unauthorized software, and information systems shall be monitored.   1. Methods of anonymizing information such as data and genetic information (selection of two or more options is possible, but an explanation should be provided)     　　　□　Retrievable anonymization will be performed.    　 ■ Retrievable anonymization will be performed.  　　　 　 Specific methods of protecting information including personal information  The individual concerned shall be given a new number or code for identification purposes. Correspondence tables linking anonymized codes with patient personal information and research results organized by anonymous codes that do not contain any personally identifiable information shall be stored separately. Only staff at affiliated institutions, research staff, and the lead researcher may view and update correspondence tables. The taking of correspondence tables and research results outside the laboratory shall be prohibited.    　　　□　No anonymization will be performed.  <Note: According to the ethical guidelines, anonymization of data or genetic information shall not be performed if this is acknowledged in the study plan that has been approved by the head of the research institution, consented to by the data provider or their legal representative, and approved by the ethical review board.>     1. Reasons for not anonymizing information 2. Explanation for not anonymizing information and overview of consent (attach consent forms) 3. Specific methods of protecting information including personal information            1. Measures for computers used to process personal information in the case that computers are used   　　　　　Correspondence tables linking anonymized codes with patient personal information and research results organized by anonymous codes that do not contain any personally identifiable information shall be stored on separate computers disconnected from the network. A record shall be kept of any modifications made to electronic data (date of modification, name of modifier). System security shall be managed and data appropriately backed up.    　(3)　Provision of personal information to entities outside of the laboratory with which the lead researcher is affiliated 　<In the ethical guidelines, anonymization of data is specified as a general rule>  　　　　□　No  ■　Yes  Anonymization　□　No  ■　Yes  Personal information protection measures    □　Research division on campus    ■　Outside institution    If the method of anonymization for data or genetic information differs from those described above in (1), this method shall be described.  If information is provided without conducting anonymization, the reason and consent for this shall be included. The relevant consent form shall also be attached. | | | | |
| ＃☆14. Providing or consigning data and information on data to outside research institutions or outsourcing contractors  Provision or consignment of data   - No   ■　Yes  ↓  　(1) Possibility of providing or consigning data to outside research institutions and names of provision destinations and outsourcing contractors  　　　□　Provision of data to outside research institutions 　　　→(go to (2)–(3))  　　　　　　Name of institution to receive data　(　　　　　　　　　　　　　　　　　　　　　　　)  　　　■　Outsourcing contractors　　　　　　　→(go to (2))  　　　　　　Name of contractor　(　　　Kyouritsu Jyuzen Hospital Testing Department　　　　　)  　　　□　Human cell, tissue, and gene back →(go to (4))  　　　　　　Name of bank　(　　　　　　　　　　　　　　　　　　　　　)  　(2) Handling of information that includes personally identifiable information during provision or consigning of data (attach any contracts made)  　　　■　Anonymization  　　　　　　①　Type of anonymization　　　　□　Non-retrievable anonymization  　　　　　　　　　　　　　　　　　　 ■　Retrievable anonymization  　　　　　　②　Specific method of anonymization  　　　　　　　　A code or number shall be assigned to the individual concerned. The personal information manager shall store correspondence tables in a secure location.  　　　□　No anonymization  　　　　 　　① Reasons for not performing anonymization and specific methods of protecting information that includes personally identifiable information        ②Explanations given to subjects regarding the provision or consignment of data to outside institutions without performing anonymization and consent given by subjects (attach any consent forms)      　　(3) The intended use of data and validity of this by institutions receiving data (excluding data provided to banks)      　　(4) Provision of data to human cell, tissue, and gene banks  　　　①　Consent to provide data to banks (attach any consent forms)    　　　②　Handling of information that includes personally identifiable information when providing data to any banks concerned  　　　　□　Anonymization  　　　　　　　Type of anonymization　　　　□　 Non-retrievable anonymization  　　　　　　　　　　　　　　　　　 □　 Retrievable anonymization  　　　　　　　Specific method of anonymization    　　　　□　No anonymization  　　　　　　　Reasons for not performing anonymization and specific methods of protecting information that includes personally identifiable information    ③　Confirmation that non-retrievable anonymization has been performed when the banks concerned sell data for general research use    　　　④　Other matters for consideration | | | | |
| 15. Informed consent   1. Informed consent procedures and methods   Subjects shall be fully briefed on the significance, purpose, methods, and expected results of this study, any disadvantages that they may suffer, and the storage and usage data. Voluntary, written informed consent shall then be obtained from subjects and data obtained. Subjects or their legal representatives may withdraw their written informed consent at any time without suffering any disadvantage.  If obtaining written informed consent from a subject is difficult and the planned research is highly important and cannot be completed without the subject’s data, then written informed consent may be obtained from the legal representative of the subject only if the ethical review board give their approval and the head of the research institution authorizes this.  Name, job title, and affiliation of individual to conduct the briefings: Takashi Mitsushima, associate professor, Department of Rehabilitation    　Checklist:  When confirming that the following items are listed in the briefing document to be given to subjects, be sure to check the mandatory items indicated by “□” and items relevant to the research indicated by “○.”    ■ The provision of data is voluntary and subjects shall suffer no disadvantage if they do not give their consent.  　　　　　 Furthermore, subjects may withdraw their written informed consent at any time without suffering any disadvantages  ■ If a subject withdraws their consent, any relevant data and research results shall be discarded, with the exception of non-retrievable anonymized data  ■ The reason subjects were selected as data providers  ■ The significance, purpose, methods, and period of the study. (The disease being investigated, analysis methods etc. The purpose of any expected future additions or modifications. The need for research in the case of monogenetic diseases, and special remarks regarding measures to prevent disadvantages.)  ■ Expected research results and any possible risks or disadvantages to subjects (including disadvantages to social life such as social discrimination )  ■ Retrievable and non-retrievable anonymization of collected data or genetic information obtained therefrom, as well as specific methods of anonymization. An explanation will be given if no anonymization can be performed.  ■ Subjects and their legal representatives may obtain or view material regarding the study plan and research methods if they wish. In such cases, subjects and their legal representatives shall ensure that the personal information of other subjects and the originality of the research are protected.  ■ The possibility that data or genetic information collected for this study may be provided to other institutions. If information is provided to other institutions, the ethical review board shall investigate whether the handling of personal information, the name of the receiving institution, and the intended use of samples are valid.  ● Methods of anonymizing data if part of the research is outsourced (only relevant research)  ○ Matters relating to the disclosure of genetic information (only relevant research)  ■ Disclosure of information and results obtained from the research  ○ The possibility that the research results may lead to intellectual property rights such as patent rights. Envisioned jurisdiction if intellectual property rights such as patent rights are obtained (only relevant research)  ○ Genetic information obtained from data may be presented at academic conferences after being anonymized (only relevant research)  ■ Storage and usage of data  ■ Storage, usage, or methods of discarding data at the end of the study. Including the possibility that data will be used in other studies and the expected contents of these studies.  ○ The academic significance of human cell, tissue, and gene banks, the name of the organization managing the bank in question, methods of anonymizing data sent to banks, and the name of the person in charge of the bank in the case that data are provided to human cell, tissue, and gene banks and likely to be sold as general research resources (only relevant research)  ○ Information related to the use of genetic counseling. The availability of genetic counseling in the case of monogenetic diseases etc. (only relevant research)  ■ Methods of procuring research funding  ■ Compensation for providing data  ■ Conflicts of interest  ■ Name and job title of the lead researcher and information regarding who to contact in the case of any queries or complaints  ○ Other special remarks  　　　　　　　Enter any specific remarks    　(2) Difficulties obtaining written informed consent from subjects  　　　　■　No difficulties  □　Difficulties 　Reason:    <Notes: When selecting a legal representative, the individual considered capable of representing the likely intentions and interests of the subject, taking into account the subject’s customs, their family structure, and situation shall be selected from among the individuals outlined below.  1. Any pre-determined guardian, parental authority, ward, or curator  2. The spouse, adult child, parent, adult sibling or grandchild, grandparent, relative they live with, or any close relative of a living subject  3. The spouse, adult child, parent, adult sibling or grandchild, grandparent, relative they live with, or any close relative of a deceased subject>      (4) Information regarding the use of genetic counseling and the state of genetic counseling in the case that a subject has a monogenetic disease etc.    　(5) Will data or genetic information be received from other institutions implementing this research?  　　　　■　No  □　Yes  　　　　　　The content of written informed consent obtained by the research institution (attach any briefing and consent documents)    　　　　　If the answer is “yes,” briefly describe the contents of data or genetic information | | | | |
| 16. Disclosing information obtained in this study to subjects  　(1) Is the genetic information of each individual subject applicable to human genome and genetic analysis research?  　　　　■　Not applicable → (go to (2))  □　Applicable  　　　　　　　① Response if the subject themself wishes to disclose information (including after the study)      　　　　　　　② Response if the subject themself does not wish to disclose information (including after the study)      　　　　　　 　③ Response if someone other than the subject wishes to disclose information (including after the study)      ・Will genetic information regarding monogenetic diseases be disclosed?  □ Yes  Response if information is to be disclosed (including cooperation with medical doctors)    　(2) The disclosure of research information obtained from studies other than human genome and genetic analysis research  　　　　　　　① Response if the subject themself wishes to disclose information (including after the study)  　　　　　　　　　 As a general rule, information shall be disclosed following the prompt delivery of the documentation or request for disclosure by the subject using the method the subject has consented to.  　　　　　　　② Response if the subject themself does not wish to disclose information (including after the study)  Information shall not be disclosed.    　　　　　　　 ③ Response if someone other than the subject wishes to disclose information (including after the study)  If the subject does not consent to this, then as a general rule, information regarding the subject shall not be disclosed to anyone else. | | | | |
| 17. Use of data provided prior to implementation of the study  　　Will data received and stored prior to implementation of the study be used?  　　　□　They will be used　　　　■　They will not be used  　　↓  　　　　　Means of protecting personal information  (1) Types and amounts of the data in question 　　　　　Type　　　　　　　　　　　　　　　Amount      (2) Period in which the data in question were gathered 　　　　　　Year　　　Month　　–　　　　　Year　　　Month  (3) Classification of data according to the conditions of consent during the period in which they were collected  　　□ Group A data [Data for which consent was given for use in the present study at the time of collection]  (attach copies of the consent forms)  　　　　① Will these data be used within the scope of the agreement?　→　□ Yes 　　　　□ No     1. State of explanations given at the time of Group A data collection regarding their use in human genome and genetic analysis research (to what extent were subjects made aware of this?)   　　　　　　1) Explained (mentioned) significance and study objectives of other human genome and genetic analysis research    　　　　　　2) Explained (mentioned) methods of managing and protecting personal information (including anonymization)    　　　　　　3) Other special remarks (including period in which consent was obtained)      　　□ Group B data [Data for which only consent was given for use in a study, but not the present study, at the time of collection]  (attach copies of the consent forms)  　　　　①　Method of anonymization  　　　　　　□ No possible risks or disadvantages to subjects through non-retrievable anonymization.  　　　　　　　　　□　Anonymization performed.  　　　　　　　　 □ Reasons for the possible risks or disadvantages to subjects being deemed very small in the present study    　　　　　　□ Reasons for the present study being deemed highly useful      　　　　　　　 　□ Reasons for implementation of this study by other methods being deemed virtually impossible or extremely difficult    　　　　　　　□ Other special remarks    　　　　② Use of Group B data collected after enforcement of the guidelines (April 1, 2001)  　　　　　　 1) Is the opportunity to refuse to have data used guaranteed in the study’s written informed consent?  　　　　　　　　　□　Guaranteed　　　　　□　Not guaranteed    　　　　　　 2) The state of explanations regarding the use Group B data in other studies at the time of collection in the present study, which will be implemented following retrievable anonymization of data (to what extent were subjects made aware of this?)  　　　　　　　a) Explained (mentioned) significance and purpose of other studies    b) Explained (mentioned) methods of managing and protecting personal information (including anonymization)       1. Other special remarks (including the period in which consent was obtained)  - Group C data [Data for which no consent was given for use in studies at the time of collection]     　　　　　①　Method of anonymization  　　　　　　□ No possible risks or disadvantages to subjects through non-retrievable anonymization.    □ Retrievable anonymization is performed (tick the option that applies to the reason for using these data)  □ The study using these data is necessary for the improvement of public health  □ The implementation of this study is virtually impossible by other methods  □ Obtaining consent from subjects or their legal representative is difficult  □ Due to laws and regulations  □ Other (provide a specific reason)  　　　　　② Measures to guarantee subjects or their legal representatives the opportunity to refuse to be contacted and have data used in this study while at the same time allowing information related to the implementation of this study to be published        　 ③ Use of Group C data collected after enforcement of the guidelines (April 1, 2001)  　 Reasons for deeming the use of data absolutely necessary such as when a study urgently needs to be conducted or when the sample size is limited in any human genome and genetic analysis research conducted following retrievable anonymization of data. | | | | |
| 18. The necessity of genetic counseling and the genetic counseling system  　(1)　Necessity  　　　　□　Present　(from (2) to (5))　　　　 ■　Absent (reason: the target disease is not a genetic disorder)  　(2) State of explanations regarding genetic counseling      (3) Methods of implementing genetic counseling  ＜This university’s genetic counseling team ＞  　　Professor Masato Maekawa, Department of Laboratory Medicine, Associate professor Hiroaki Miyajima, First Department of Internal Medicine, Lecturer Naomi Satou, Department of Clinical Nursing      　(4) The genetic counseling system and the names, job titles, affiliations, and roles of the staff      　(5) Genetic counseling implementation methods if genetic information regarding monogenetic diseases is to be disclosed  　　　(including cooperation with medical doctors) | | | | |
| 19. Storage methods and need for data 　　　※Data include treatment information (clinical assessments etc.)   1. Storage of data during the study period, storage period, storage method, and need for storage     ■ Stored  　　　Need for storage　　Stored to obtain results regarding primary and secondary endpoints.  Storage method　　Blood shall be stored in freezers at −80°C.  　　　　　　　　　　　 Personal information in questionnaires and assessment results shall be anonymized and stored on a computer.  Storage period　　　Until tests are completed.    　　□ Not stored (discarded)     1. Storage of data at the end of the study period, storage period, storage method, need for storage, and the state of explanations and consent regarding this   ■ Not stored (discarded)  □ Stored  Need for storage  Storage method    Storage period  State of explanations and consent    　(3) Possibility of data being used in other studies, expected content of these studies, and explanations and consent regarding this    Data will not be used in other studies. | | | | |
| 20. Methods of anonymizing and discarding data 　　※Data include treatment information (clinical assessments etc.)   1. Methods of anonymizing and discarding data at this university during the study period   ■ Absent (storage)  □ Present  　　　Disposal methods    　　　Anonymization methods   1. Methods of anonymizing and discarding data at this university at the end of the study period   ■ Present  　　　Disposal methods　　Blood shall be disposed of with the usual care.  　　　　　　　　　　　　Data stored on computers shall be electronically discarded.  　　　Anonymization　 　　Correspondence tables linking personal information with codes shall be electronically discarded and specimens and electronic data shall undergo retrievable anonymization.  　　□ Absent (storage)  　　　Need for this    　(3) Methods of anonymizing and discarding data and research results when written informed consent is withdrawn    　　The data of the patient (subject) in question shall be removed from any correspondence tables linking personal information with codes. Code labels shall be peeled off frozen blood samples and these samples discarded immediately with the usual care. Data in paper form shall be immediately disposed of by use of a shredder. Data stored on computers shall be immediately disposed of electronically. | | | | |
| ＃＊21. Publication and release of research results  Subjects’ privacy shall be protected when results are released. | | | | |
| 22. Methods of procuring research funding  The Department of Rehabilitation shall bear all costs related to tests. | | | | |
| 23. Compensation  　□　Yes　　　Please provide specific details if the answer is “yes”  　■　No | | | | |
| 24 Conflicts of interest  　□　Yes　　　Please provide specific details if the answer is “yes”  　■　No | | | | |
| 25. Predictions regarding the publication of research results on undisclosed items and reasons for not disclosing these items when publishing the summary of proceedings of this review 　　※If you do not wish to have the summary of proceedings published, please note this below. If there are no items that should not remain undisclosed, please write “not applicable” in the space below.  (1) Reasons for nondisclosure (please tick those that apply from the following options)  　□ There is risk that the human rights of the subject, their blood relatives, or family may be violated  Not applicable    　□ There is risk that intellectual property rights such as patent rights or the originality of this research may be violated  Not applicable  　□ Other (please specify)  Not applicable  (2) Predictions regarding the publication of research results on undisclosed items  Not applicable | | | | |
| 26. Other matters of note | | | | |
| 27. Who to contact if subjects have any queries or complaints regarding this study (name, job title, affiliation, telephone, and FAX)    Takashi Mitsushima, associate professor, Department of Rehabilitation  Tel: 053-435-2746 (Department of Rehabilitation) (weekdays 8:30–17:15)  FAX: 053-435-2746 (Department of Rehabilitation) | | | | |
