# Supplementary material for: Effects of Home-Based Interval Walking Training on Thigh Muscle Strength and Aerobic Capacity in Female Total Hip Arthroplasty Patients: A Randomized, Controlled Pilot Study
Source: PLoS One. 2014 Sep 30;9(9):e108690. doi: 10.1371/journal.pone.0108690 (PMC4182539; doi:10.1371/journal.pone.0108690)
Supplement: Medical Research Ethics Review Application Form S2 — Medical Research Ethics Review Application Form in original language (in Japanese). (DOC) [file pone.0108690.s005.doc]

医 学 研 究 倫 理 審 査 申 請 書

　平成21年7月14日

浜松医科大学長　　殿

　　　　　　　　　　　　　　　　　　　　　　　研究責任者　所　　属 リハビリテーション科

　　　　　　　　　　　　　　　　　　　　　　　　　　　　　　　　職・氏名　准教授　美津島　隆　　印

　　　　　　　　　　　　　　　　　　　　　　　所属職域の責任者　職・氏名　准教授　美津島　隆　　印

※受付番号

| １　研究課題名 | | | 人工股関節置換術後患者の在宅における新しいトレーニング方法の開発 | |
| --- | --- | --- | --- | --- |
| ２　研究代表者 | | | 氏名　美津島　隆　　所属　リハビリテーション科　　　職名　准教授 | |
| ３　研究組織 | | |  | |
|  |  | ３－１  学内 | 研究責任者・研究担当者の所属・職・氏名 | 役　　　　　割 |
|  |  |  | リハビリテーション科  准教授　美津島　隆  リハビリテーション科  助教　山内　克哉  リハビリテーション部  理学療法士　　森島　優 | 研究の統括  医学的管理、測定・記録  データ解析  測定・記録、介入方法の指導 |
|  | 学外共同研究機関  ・企業 | ３－２  本学の倫理規定、倫理委員会決定に従う場合 | 学外研究者の所属・職・氏名 | 役　　　　　割 |
|  |  |  | 信州大学大学院医学系研究科  教授　能勢　博 | 介入方法の指導 |
|  |  | ３－３  独自に倫理委員会を有する場合 | 学外共同研究機関名、企業名・研究担当者の氏名 | 役　　　　　割 |
|  |  |  |  |  |
| ４　本研究における個人情報の分担管理者  （本研究における個人情報の分担管理者を当該研究の研究責任者又は研究担当者が兼ねることはできない） | | | 所属　　リハビリテーション科  職　　　医員  氏名　　入澤　寛 | |
| ５　研究等の目的 | | | 松本市熟年体育大学において健常高齢者に対する効果が報告されている、「インターバル速歩トレーニング」を、人工股関節置換術後患者の在宅トレーニングに応用し、運動機能向上、健康増進に対する有効性を検討する。 | |
| ６　研究の概要等 | | | 概要:  人工股関節置換術後患者を対象とし、無作為に2群に割り付ける（インターバル速歩群、コントロール群）。インターバル速歩群には最大歩行速度の70％の速歩と30％緩歩を繰り返す、インターバル速歩トレーニングの実施を指導する。コントロール群には、通常通りの生活を維持するように指導する。介入期間は12週間とし、前後で、その効果を筋力、運動耐容能（最大酸素摂取量、無酸素性作業閾値）、身体活動量から検証する。また、歩行満足度、健康関連QOLといった社会心理学的機能面からも検証する。さらに前後で股関節痛の変化も調査する。 | |
|  |  |  | 研究等の対象（研究協力者）：　人工股関節置換術後患者30名  実施場所：浜松医科大学医学部附属病院リハビリテーション部  予測症例（検体）数：　30症例  提供を受けようとする１症例（検体）当たりの試料等の種類と量：  ※種類と量の行は必要に応じて追加すること。なお、試料には診療情報（臨床評価表など）も含む。  種類　血液　　　　 　　　量　　10　ml  種類　臨床評価表　 　　　量　　2　枚 | |
| 7　研究期間  　(3年を限度とし､それ以上に及ぶときは､継続申請すること｡ただしヒトゲノム・遺伝子解析研究については5年を限度とする。) | | | 平成　21　年　7　月　から　平成　23　年　7　月　まで | |
| 8　共同研究の場合、共同研究機関における承認の状況等  (1)　当該研究計画の承認の状況  共同研究機関については本倫理委員会において承認された方法・手続きにすべて従う。    (2)　インフォームド・コンセントの状況    信州大学大学院 能勢教授からは本研究責任者より書面および口頭にて十分に説明した後、  書面にて同意を得ている。  (3) 匿名化の状況  有 　　連結可能匿名化による。結果の解析を始める前に資料から住所や氏名など個人を特定できる情報を削り、符号を付けて匿名化する。匿名化した符号と患者の個人情報とを連結させる対応表は、個人情報分担管理者が厳重に保管する。また、資料や診療記録、個人情報も厳重に別々に保管する。  <参考：提供者又は代諾者等が同意し、かつ倫理審査委員会の承認を受け、研究を行う機関の長が許可した研究計画書において認められている場合には、試料等又は遺伝情報の匿名化を行わないとしている。>  (4)　その他の特記事項  (5) 海外との共同研究が含まれる場合は、その国名及び我が国の指針に相当する法・基準等の概略と対応策等 | | | | |
| ☆9　研究の実施による結果とその開示・公表に関する考え方 | | | | |
| 10試料等提供者、被験者または研究協力者を選ぶ方針（試料等の提供が他の研究機関からのみの場合を除く。）  (１)　提供者等が、疾病や薬剤反応異常等を有する場合及びその可能性がある場合に該当するか。  　　　　■　該当する。  　　その病名又はそれに相当する状態像など  　　　　　人工股関節置換術後  □　該当しない。    (２)選択に当たって合理性・正当性・公平性が確保されているか。（合理的に選択していることがわかるよう具体的な方法を記入）  　　　　 試料などの提供者に対する医学的・精神的影響及びそれらに配慮した研究方法の是非等について慎重に検討する。特に、インフォームド・コンセントの手続及び方法、個人情報の保護の方法、研究により予測される結果及びその開示の考え方、試料等の保存及び使用の方法並びに遺伝カウンセリングの考え方について明確に説明する。また、試料などの提供者が、人工股関節置換術後という疾病を有するため、該当する病名又はそれに相当する状態像等を告知する。その上で、該当する患者に当研究に関して患者用説明文書を用いて十分に説明し、インフォームドコンセントが得られれば、研究を開始する。 | | | | |
| ＃☆11　提供者・被験者および家族等に対する利益および危険や不利益（社会的な差別を含む）の予測及びその防止のための方法等についての配慮  　（提供者・被験者が、治療又は予防方法が確立していない単一遺伝子疾患であって、精神・知的障害を伴うものである場合には、研究の必要性、当該提供者に対する医学的・精神的影響及びそれらに配慮した研究方法の是非等に係る検討内容を含む。）  （1）用いる研究手法は既に確立されたものであるか。    以下のように、用いる研究手技は既に確立されたものである。  採血は静脈より行い、危険性はほとんどない。  筋力測定は非侵襲的であり、手技的にも容易で危険性はほとんどない。  運動耐用能測定は、医師の十分な監視のもと厳なモニタリング下で実施するため危険性はほとんどない。  （2）予測される当該個人および家族への利益あるいは不利益及び危険性はあるか。  群間比較する場合は、各群における利益あるいは不利益及び危険性を他群のそれと比べて具体的に記すこと。またその根拠となる添付文書、文献等を添付すること。  　利益：身体機能面において筋力・運動耐容能向上、精神機能面から歩行満足・健康関連QOL改善が期待できる。  不利益：運動トレーニングによる筋肉痛、関節痛、また転倒による捻挫や骨折等。  （3）有害事象が生じた場合の対応  　有害事象が生じた場合は適切な治療を行う。重篤と判断される有害事象については直ちに病院長に報告を行う。  （4）研究等が自然環境に及ぼす影響と安全性が確保されているか。  　　試料はすべて適切な方法で処理、廃棄されるため、自然環境に及ぼす影響はなく、安全性も確保されている。 | | | | |
| ＊12　研究協力者個人および家族等に対する危険や不利益（社会的な差別を含む）の予測及び人権の擁護  （1）予測される当該個人および家族への不利益及び危険性はあるか。  (2) 研究協力者個人および家族等が危険や不利益を受けない権利  (3) 研究協力者個人および家族等の自己決定の権利 | | | | |
| １3　当該研究における個人情報の保護の方法  個人情報の漏えい、消失またはき損の防止その他個人情報の安全管理のため、以下の措置を講じる。組織的安全管理措置として、安全管理についての研究者などの責任と権限を明確に定め、安全管理を厳重に行い、その実施状況を確認する。人的安全管理措置として、研究者などに対する、業務上秘密とされた個人情報の非開示契約の締結について確認する。物理的安全管理措置として、入退室（館）の管理、個人情報の盗難の防止などをおこなう。技術的安全管理措置として、個人情報およびそれを取り扱う情報システムのアクセス制御、不正ソフトウエア対策、情報システムの監視をおこなう。   1. 試料等の情報、遺伝情報の匿名化の方法等　（２つ以上選択可。ただし、その場合は要説明）     　　　□　連結不可能匿名化をする。    　 ■　連結可能匿名化をする。  　　　 　個人情報を含む情報の保護についての具体的方法  必要な場合に個人を識別できるよう当該個人に新たに符号又は番号を付す。匿名化した符号と患者個人情報との対応表と、匿名化コードで整理され個人を特定できる情報を含まない研究結果は、それぞれ別々に保管する。対応表は所属職域の責任者・研究責任者・研究担当者のみが閲覧、更新できることとする。対応表、研究結果ともに研究室外への持ち出しは禁止する。    　　　□　匿名化しない。  <参考：倫理指針では、提供者又は代諾者等が同意し、かつ、倫理審査委員会の承認を受け、研究を行う機関の長が許可した研究計画書において認められている場合には、試料等又は遺伝情報の匿名化を行わないとしている。>  　　　 ①　匿名化しない理由      　　　　②　匿名化を行わないことについての説明、同意等の概略　(同意書を添付のこと｡)       1. 個人情報を含む情報の保護についての具体的方法            1. コンピュータを利用する場合は、個人に関する情報を処理するコンピュータに関する措置   　　　　　匿名化した符号と患者個人情報との対応表と、匿名化コードで整理され個人を特定できる情報を含まない研究結果は、それぞれ別々のネットワークから切り離されたコンピュータに保管する。電子データの修正を行う場合、データ修正（修正日、修正者）の記録を残す。システムのセキュリティを管理、適切にデータのバックアップを行う。    　(3)　研究責任者の所属する研究室以外への個人情報の提供　<倫理指針では原則として試料等の匿名化を明記している>  　　　　□　無  ■　有  匿名化　□　無  ■　有  個人情報保護の方策    □　学内の研究部門である。    ■　学外の機関である。    試料等又は遺伝情報の匿名化の方法等が上記(1)と異なる場合はその概略を記入のこと。  なお、匿名化を行わずに提供するときは、その説明、同意等を含む。また、その同意書を添付のこと | | | | |
| ＃☆14　試料及び試料に関わる情報の学外の研究組織または業務外部委託先への提供または委託  試料等の提供または委託   - 無   ■　有  ↓  　(1)学外の機関組織への提供及び業務委託の可能性並びにその提供先又は受託者等  　　　□　学外の研究組織への提供　　　→（(2)～(3)へ）  　　　　　　提供先の組織名　（　　　　　　　　　　　　　　　　　　　　　　　）  　　　■　業務の外部委託　　　　　　　→（(2)へ）  　　　　　　受託者名　（　　　協立十全病院検査部　　　　　）  　　　□　ヒト細胞・遺伝子・組織バンク→（(4)へ）  　　　　　　バンク名　（　　　　　　　　　　　　　　　　　　　　　）  　(2)提供時又は業務委託時の個人識別情報が含まれている情報の取扱い(契約する場合は､契約書を添付のこと｡)  　　　■　匿名化する。  　　　　　　①　匿名化の種類　　　　□　連結不可能匿名化  　　　　　　　　　　　　　　　　　　■　連結可能匿名化  　　　　　　②　匿名化の具体的方法  　　　　　　　　当該個人に符号又は番号を付す。対応表は、個人情報分担管理者が厳重に保管する。  　　　□　匿名化しない。  　　　　　　①　匿名化できない理由、個人識別情報が含まれている情報の保護の具体的方法等         1. 匿名化を行わずに外部の機関に提供又は委託することについての試料等提供者等への説明、同意の状況等（同意文書を添付のこと。）       　　(3)　提供する場合における提供先の利用目的及びその妥当性（バンクへの提供を除く。）      　　(4)　ヒト細胞・遺伝子・組織バンクへの試料等の提供  　　　①　バンクに提供することについての同意事項等（同意文書を添付のこと。）    　　　②　当該バンク提供時の個人識別情報が含まれている情報の取扱い  　　　　□　匿名化する。  　　　　　　　匿名化の種類　　　　□　連結不可能匿名化  　　　　　　　　　　　　　　　　　□　連結可能匿名化  　　　　　　　匿名化の具体的方法    　　　　□　匿名化しない。  　　　　　　　匿名化できない理由、個人識別情報が含まれている情報の保護の具体的方法等    　　　③　当該バンクが当該試料等を一般的な研究用試料等として分譲する際に連結不可能匿名化がなされることの確認の状況等    　　　④　その他参考となる事項 | | | | |
| 15　インフォームド・コンセントについて   1. インフォームド・コンセントのための手続き及び方法   提供者に対して、その研究の意義、目的、方法、予測される結果、提供者が被るおそれのある不利益、試料等の保存及び使用方法等について十分な説明を行った上で、自由意思に基づく文書による同意（インフォームド・コンセント）を得て、試料等の提供を受ける。提供者又は代諾者等は、インフォームド・コンセントを、いつでも不利益を受けることなく文書により撤回することができることを確認する。  提供者からインフォームド・コンセントを受けることが困難な場合には、その実施しようとしている研究の重要性が高く、かつ、その人からの試料等の提供を受けなければ研究が成り立たないと倫理審査委員会が承認し、研究を行う機関の長が許可した場合に限り、提供者の代諾者等からインフォームド・コンセントを受ける。  説明者の所属・職・氏名　　リハビリテーション科　准教授　美津島　隆    　チェックリスト：  被験者等への説明文書に以下の項目が記載されていることを確認するため、□は記載必須項目なので必ずチェックすること。○は該当する研究については必ずチェックを入れること。    　　　 ■　試料等の提供は任意であり、提供に同意しなくても不利益な対応を受けることはない。  　　　　　 また、いつでも不利益を受けることなく同意を文書により撤回できること  　　　 ■　同意が撤回された場合には、当該撤回に係る試料等及び研究結果が連結不可能匿名化されている場合等を除き、廃棄されること  　　　 ■　提供者として選ばれた理由  　　　 ■　研究の意義、目的及び方法（対象とする疾患、分析方法等。将来の追加、変更が予想される場合はその旨。単一遺伝子疾患等の場合には研究の必要性、不利益を防止するための措置等の特記事項等｡)、期間  　　　 ■　予測される研究結果及び提供者等に対して予測される危険や不利益（社会的な差別等社会生活上の不利益を含む。）  　　　 ■　提供を受けた試料等又はそれから得られた遺伝情報についての連結可能匿名化又は連結不可能匿名化の別、及び匿名化の具体的方法。匿名化できない場合にあっては、その旨及び理由  　　　 ■　提供者及び代諾者等の希望により、研究計画及び研究方法についての資料を入手又は閲覧することができる。その場合、他の提供者等の個人情報の保護や研究の独創性の確保に支障が生じない範囲内であることが遵守されていること。  　　　 ■　試料等又はそれから得られた遺伝情報を他の機関へ提供する可能性の有無。提供する場合は、倫理審査委員会により、個人情報の取扱い、提供先の機関名、提供先における利用目的が妥当であることについて、審査されていること  　　　 ●　研究の一部を委託する場合、試料等の匿名化の方法等（該当する研究のみ）  ○ 遺伝情報の開示に関する事項（該当する研究のみ）  ■ 研究で得られた情報・結果の開示について  ○　将来、研究の成果が特許権等の知的財産権を生み出す可能性があること。特許権等の知的財産権を生み出した場合の想定される帰属先（該当する研究のみ）  　　　 ○　試料等から得られた遺伝情報は、匿名化された上、学会等に公表され得ること（該当する研究のみ）  　　　 ■　試料等の保存及び使用方法  　　　 ■　研究終了後の試料等の保存、使用又は廃棄の方法。他の研究への利用の可能性の有無と予測される研究内容を含む。  　　　 ○　試料等をヒト細胞・遺伝子・組織バンクに提供し、一般的に研究用資源として分譲することがあり得る場合には、バンクの学術的意義、当該バンクを運営している機関の名称、提供される試料等の匿名化の方法及びバンクの責任者の氏名（該当する研究のみ）  　　　 ○　遺伝カウンセリングの利用に係る情報。単一遺伝子疾患等の場合には、遺伝カウンセリングが利用可能であること等（該当する研究のみ）  　　　 ■　研究資金の調達方法について  ■ 試料等の提供についての補償  ■ 利害の衝突  　　　 ■　研究責任者の氏名及び職名、問い合わせ、苦情等の窓口の連絡先等に関する情報  　　　 ○　その他の特記事項  　　　　　　　具体的な特記事項を記入    　(2)　提供者本人からインフォームド・コンセントを受けることの困難性。  　　　　■　困難でない。  □　困難である　その理由：    <参考：代諾者等を選定する考え方としては、以下に定める人の中から、提供者の家族構成や置かれている状況、慣習等を勘案し等を勘案し、提供者の推測される意思や利益を代弁できると考えられる人を選定する。  １．任意後見人、親権者、後見人や保佐人が定まっているときはその人  ２．提供者の配偶者、成人の子、父母、成人の兄弟姉妹若しくは孫、祖父母、同居の親族又はそれらの近親者に準ずると考えられる人  ３．死亡した提供者の配偶者、成人の子、父母、成人の兄弟姉妹若しくは孫、祖父母、同居の親族又はそれらの近親者に準ずると考えられる人>      (4)　提供者が単一遺伝子疾患等の場合は、遺伝カウンセリングの利用に関する情報の説明及び遺伝カウンセ  　　　リングの状況    　(5)　他の研究実施機関から試料等又は遺伝情報の提供を受けることの有無  　　　　■　無  □　有  　　　　　　その研究実施機関におけるインフォームド・コンセントの内容等（説明･同意文書を添付すること｡）    　　　　　有の場合は内容を簡単に記載する | | | | |
| 16　この研究で得られた情報の提供者等への開示  　(1)　個々の提供者の遺伝情報が明らかとなるヒトゲノム・遺伝子解析研究に該当の有無  　　　　■　該当しない。→（（２）へ）  □　該当する。  　　　　　　　①　提供者本人が開示を希望する（事後を含む）場合の対応      　　　　　　　②　提供者本人が開示を希望していない（事後を含む）場合の対応      　　　　　　　③　提供者以外が開示を希望する（事後を含む）場合の対応      ・単一遺伝子疾患等に関する遺伝情報の開示を行うか  □有  開示しようとする場合の対応（診療担当医師との連携を含む）    　（2)　 ヒトゲノム・遺伝子以外の得られた研究情報の開示について  　　　　　　　①　提供者本人が開示を希望する（事後を含む）場合の対応  　　　　　　　　　原則として、被験者に対し、遅滞なく書面の交付または開示の求めを行った者が同意した方法で開示する。  　　　　　　　②　提供者本人が開示を希望していない（事後を含む）場合の対応  開示しない。    　　　　　　　③　提供者以外が開示を希望する（事後を含む）場合の対応  提供者の同意がない場合には、提供者の情報を、提供者以外の人に対し、原則として開示しない。 | | | | |
| 17　研究実施前提供試料等の利用  　　本研究の実施前に提供され、保存されている試料等を利用するか否か。  　　　□　利用する。　　　　■　利用しない。  　　↓  　　　　　個人情報の保護についての方法  (1)　当該試料等の種類及び量　　　　　種類　　　　　　　　　　　　　　　量      (2)　当該試料等が集められた時期　　　　　　年　　　月　　～　　　　　年　　　月  (3)　当該試料等が集められた時点における同意の状況に応じた区分  　　□　Ａ群試料等【提供時に当該研究における利用を含む同意が与えられている試料等】  （同意文書の写しを添付すること）  　　　　①　その同意の範囲内の利用か　→　□　範囲内の利用　　　　□　範囲外の利用となる。     1. 当該Ａ群試料等が提供された時点に行った他のヒトゲノム・遺伝子解析研究への利用に関する説明の状況（言及の程度）等   　　　　　　1)　説明（言及）した他のヒトゲノム・遺伝子解析研究の意義、研究目的等    　　　　　　2)　説明（言及）した個人に関する情報の管理・保護（匿名化等を含む）の方法等    　　　　　　3)　その他特記事項（同意が得られた時期などを含む）      　　□　Ｂ群試料等【試料等の提供時に、当該研究での利用が明示されていない研究についての同意のみが与えられている試料等】  （同意文書の写しを添付すること）  　　　　①　匿名化の方法  　　　　　　□　連結不可能匿名化されていることにより、試料等の提供者等に危険や不利益が及ぶ可能性はない。  　　　　　　□　連結可能匿名化されている。  　　　　　　　　□　本研究により試料等の提供者等に危険や不利益が及ぶ可能性が極めて少ないと認められる理由      　　　　　　　　□　研究の有用性が高度と認められる理由      　　　　　　　　□　他の方法では実際上研究の実施が不可能又は極めて困難と認められる理由    　　　　　　　　□　その他の特記事項    　　　　②　指針施行（平成13年4月1日）後に提供されたＢ群試料等の利用の場合  　　　　　　1)　本研究に関するインフォームド・コンセントにおいて、試料等の利用を拒否する機会の保障  　　　　　　　　　□　保障されている。　　　　　□　保障されていない。    　　　　　　2)　連結可能匿名化の上で実施される研究において、当該Ｂ群試料等が提供された時点に行った他の研究への利用に関する説明の状況（言及の程度）等  　　　　　　　a) 説明（言及）した他の研究の意義、目的等    b)　説明（言及）した個人に関する情報の管理・保護（匿名化等を含む）の方法等       1. その他特記事項（同意が得られた時期などを含む）  - Ｃ群試料等【提供時に研究に利用することの同意が与えられていない試料等】     　　　　　①　匿名化の方法  　　　　　　　□ 連結不可能匿名化されていることにより、試料等の提供者等に危険や不利益が及ぶ可能性はない。    □ 連結可能匿名化されている（その試料等を用いる理由で該当するものをチェックすること）  □その試料等を用いた研究が公衆衛生の向上のために必要である。  □他の方法では事実上、研究の実施が不可能である。  □提供者又は代諾者等の同意を得ることが困難である  □法令に基づく場合  □その他（具体的な理由を記載すること）  　　　　　②　研究の実施状況について情報の公開を図り、併せて提供者又は代諾者等に問い合わせ及び試料等の研究への利用を拒否する機会を保障するための措置        　 ③　指針施行（平成13年4月1日）後に提供されたＣ群試料等の利用の場合  　 ヒトゲノム･遺伝子解析研究において、連結可能匿名化の上で実施される研究においては、緊急に  研究を実施する必要があり、かつ症例数が限られているなど、真にやむを得ないと認められる理由 | | | | |
| 18　遺伝カウンセリングの必要性及びその体制等  　(1)　必要性の有無  　　　　□　有　（（2）～（5）へ）　　　　■　無（理由：対象は遺伝的に関与する疾患ではないため）  　(2)　遺伝カウンセリングについての説明の状況      (3)　遺伝カウンセリングの実施方法  ＜本学の遺伝カウンセリングチーム＞  　　臨床検査医学　　前川　真人　教授、内科学第一　　宮嶋　裕明　准教授、臨床看護学　　佐藤直美　講師      　(4)　遺伝カウンセリングの体制、担当者の所属・職・氏名及び役割      　(5)　単一遺伝子疾患等に関する遺伝情報を開示しようとする場合は、遺伝カウンセリングの実施方法等  　　　（診療担当医師との連携を含む。） | | | | |
| 19　試料等の保存方法及びその必要性等　　　※試料には診療情報（臨床評価表など）も含む   1. 研究期間中の保存の有無、必要性、保存期間、保存の方法等     ■有  　　　保存の必要性　　主要・副次評価項目についての結果を得るために保存する。  保存法　　　　　血液は−80℃のフリーザーに保存する。  　　　　　　　　　　　問診票・評価測定結果は個人情報を匿名化し、コンピュータに入力保存する。  保存期間　　　　試験の終了時までとする。    　　□無（廃棄）     1. 研究期間終了後の保存の有無、保存期間、保存の方法及び必要性並びに説明・同意の状況等   ■無（廃棄）  □有  保存の必要性  保存法    保存期間  説明・同意の状況等    　(3)　他の研究への利用の可能性と予測される研究内容及びこれに関する説明・同意の状況等    他の研究への利用の可能性はない。 | | | | |
| 20　試料等の廃棄方法及びその際の匿名化の方法　　※試料には診療情報（臨床評価表など）も含む   1. 研究期間中の本学での廃棄方法及びその際の匿名化の方法等   ■無（保存）  □有  　　　廃棄方法    　　　匿名化の方法   1. 研究期間終了後の本学での廃棄方法及びその際の匿名化の方法等   ■有  　　　廃棄方法　　　　　血液は通常診療に準じた方法にて廃棄する。  　　　　　　　　　　　　コンピュータに入力保存したデータは電子的に廃棄する。  　　　匿名化の方法　 　　個人情報と符号との対応表を電子的に廃棄して、検体や電子データを連結不可能匿名化する。  　　□無（保存）  　　　その必要性    　(3)　インフォームド・コンセントの撤回があった場合の試料等及び研究結果の匿名化、廃棄の方法等    　　個人情報と符号との対応表から当該患者のデータを削除する。フリーザーに保存した血液は符号のついたラベルをはがした上で通常診療に準じた方法で直ちに廃棄する。  紙媒体のデータは直ちにシュレッダーにより廃棄する。コンピュータに入力保存したデータは直ちに電子的に廃棄する。 | | | | |
| ＃＊21　研究結果の公開、公表  公表の際には、被験者のプライバシーを保全する。 | | | | |
| 22　研究資金の調達方法  全ての検査に関わる費用は、リハビリテーション科研究費で支払う。 | | | | |
| 23　補償の有無  　□　有　　　有の場合は具体的に記すこと  　■　無 | | | | |
| 24　利害の衝突の有無  　□　有　　　有の場合は具体的に記すこと  　■　無 | | | | |
| 25　本審査に係る議事要旨の公開に当たり、非公開とすべき理由および非公開とする事項に関する研究成果の  社会への公表の見通し等　　※議事要旨の非公開を希望する場合は、以下記入すること。非公開とすべき事項がない場合は、空欄に該当なしと記入すること  （1）非公開とすべき理由（下記のうち該当するものにチェックすること）  　□　試料等提供者、その血縁者又は家族の人権の保護に支障が生じるおそれがある  該当なし    　□　研究に係る独創性又は特許権などの知的財産権の保護に支障が生じるおそれがある  該当なし  　□　その他（具体的に記入すること）  該当なし  （2）非公開とする事項に関する研究成果の社会への公表の見通し等  該当なし | | | | |
| 26　その他参考となる事項 | | | | |
| 27　本研究に関する提供者等の問い合わせや苦情等の窓口となる者（所属・職・氏名・電話・FAX 等）    リハビリテーション科　准教授　美津島　隆  　　電話：０５３－４３５－２７４６（リハビリテーション科）（平日８：３０〜１７：１５）  FAX ：０５３－４３５－２７４６（リハビリテーション科） | | | | |
